# The Role of Prosody in Expressing Subjective and Objective Causality in English

**DOI:** 10.1177/00238309251369482

**Published:** 2025-11-02

**Authors:** Na Hu, Aoju Chen, Hugo Quené, Ted J. M. Sanders

**Affiliations:** Department of Languages, Literature and Communication, Institute for Language Sciences, Utrecht University, The Netherlands

**Keywords:** Prosody, causality, subjectivity, production

## Abstract

Numerous studies have established that prosody plays an important role in expressing meanings and functions. However, it remains unknown whether prosody is employed to convey the distinction between subjective causality (CLAIM-ARGUMENT) and objective causality (CONSEQUENCE-CAUSE). This study aimed to address this issue in English, where both types of causality are typically expressed using the same connective. Two production experiments were conducted, focusing on causality in backward order (*Q* “because” *P*) and in forward order (*P* “so” *Q*), respectively. The results show that subjective causality exhibited a larger F0 range, less integrated prosody, and a distinctive F0 contour shape compared with objective causality. These findings highlight the role of prosody in expressing subjective and objective causality in the absence of explicit lexical markers in English.

## 1 Introduction

Causality is a fundamental concept in language and cognition (Graesser et al., 1994; Sanders & Spooren, 2015; Sanders & Sweetser, 2009; Singer et al., 1994; Stukker et al., 2008). Various categorizations of causal relations have been proposed, such as content, epistemic, and speech act distinctions (Sweetser, 1990), as well as subject matter versus presentational relations (Mann & Thompson, 1988; for an overview, see Sanders, 1997; Sanders & Spooren, 2009). In this study, we focus on one specific categorization: the objective–subjective distinction (Pander Maat & Sanders, 2000). Although existing discourse studies have extensively examined the expression of these two types of causality, the majority of these studies have predominantly focused on lexical means, with little attention to the role of prosody. This study aimed to address this research gap by exploring the role of prosody in conveying subjective and objective causality.

Causality is often expressed in complex or compound sentences that consist of an “antecedent” *P* and a “consequent” *Q* connected by a causal connective. The order of *P* and *Q* indicates the order of causality (Sanders et al., 1992): forward order when *P* precedes *Q* (as in Sentences [1] and [2] in Table 1), and backward order when *Q* precedes *P* (as in Sentences [3] and [4] in Table 1).

**Table 1.** Different Orders and Types of Causality.

		Order of causality	
		Forward *P* (C1) → *Q* (C2)	Backward *Q* (C1) ← *P* (C2)
Type of causality	Objective	[1] Heidi won first prize at the art festival so she is thrilled.	[3] Heidi is thrilled because she won first prize at the art festival.
	Subjective	[2] Heidi won first prize at the art festival so she must be talented.	[4] Heidi must be talented because she won first prize at the art festival.

*Source*. Examples adopted from Traxler et al. (1997).

Causality between *P* and *Q* can be constructed in two distinct ways. One way is through the reasoning of a thinking subject, such as a speaker, an author, or a third-person character engaged in a discourse. In this case, the “antecedent” *P* introduces an actual event in the physical world (e.g., “*Heidi won first prize at the art festival*” as shown in Sentences [2] and [4] in Table 1), whereas the “consequent” *Q* represents an inference or opinion (e.g., “*Heidi must be talented*” as in Sentences [2] and [4] in Table 1; Sanders et al., 1992). The other way to establish causality involves reporting events happening in the physical world, with *P* stating a cause and *Q* a consequence (e.g., “*Heidi is thrilled*” as in Sentences [1] and [3] in Table 1). When causality is constructed through the reasoning of a thinking subject, it is considered subjective (Sanders & Spooren, 2015; Sanders & Sweetser, 2009); otherwise, it is categorized as objective. Other terms used to describe this distinction include “pragmatic” versus “semantic” (Sanders et al., 1992), “internal” versus “external” (Halliday & Hasan, 1976), “diagnostic” versus “simple-causal,” and “presentational” versus “subject matter” (Mann & Thompson, 1988), “epistemic” versus “content” (Sweetser, 1990), “explanation” versus “justification” (Beebee, 2018; Nølke, 1995), and “cause” versus “reason” relations (Blochowiak et al., 2020).

The distinction in construction gives rise to several cognitive differences between subjective and objective causality. First, subjective causality involves the inclusion of a cognitive entity, commonly referred to as the “Subject of Consciousness” (SoC), whereas objective causality does not. Second, the involvement of an SoC renders subjective causality cognitively more complex compared with objective causality (Traxler et al., 1997; Zufferey & Gygax, 2015). Third, in subjective causality, where *P* pertains to the physical world and *Q* pertains to the mental world, the meanings of *P* and *Q* are relatively distant from each other, constituting two distinct separate semantic units (Zufferey, 2012). In contrast, in objective causality, both *P* and *Q* describe events occurring in the physical world, resulting in a more integrated relationship between their meanings (Sweetser, 1990; Verhagen, 2005; Zufferey, 2012).

In some languages, subjective and objective causality are expressed using different causal connectives that explicitly specify the type of causality being conveyed. For example, in Mandarin, the causal connective *kejian* (“so”) serves as a prototypical subjective causal connective, indicating to the addressees that the causal connection being conveyed is constructed through the speaker’s reasoning. Conversely, the connective *yushi* (“so”) is used in objective causality, informing the addressees that the causality being communicated concerns only events occurring in the physical world (Li et al., 2013). Similarly, in German, *denn* (“because”) and *weil* (“because”) are prototypical causal connectives expressing subjective and objective causality, respectively (Stukker & Sanders, 2012). Such specialized causal connectives are observed in other languages as well, such as *daardoor* (“so”) in Dutch, which typically marks objective causal relations (Stukker et al., 2008), and *puisque* (“since”) in French, which is a prototypical subjective causal connective (Zufferey, 2012).

However, in other languages, causal relations are often expressed using a general causal connective, even when specialized ones are available. A case in point is English, where general causal connectives such as “so” and “because” are frequently used to express causality regardless of the type, despite the existence of the specialized subjective causal connective “since” (Andersson, 2016; Couper-Kuhlen, 1996; Sweetser, 1990; Taboada, 2006). Causal connectives without a clearly attributed type of causality also include *suoyi* (“so”) in Mandarin, *parce que* (“because”) and *car* (“because”) in French (Blochowiak et al., 2020; Nazarenko, 2000; Zufferey et al., 2018).

General causal connectives do not provide explicit information about the type of causality being conveyed, making it difficult for addressees to reconstruct it (Traxler et al., 1997). This raises the question of whether other communicative means would be employed to distinguish the two types of causality. Discourse analysts have long contemplated the potential role of prosody in this regard. For example, Rutherford (1970) and Sweetser (1990) proposed that, in subjective causality, the clauses *P* and *Q* should be produced as two intonational units with a prosodic boundary between them, as they constitute two meaning units. Conversely, in objective causality, the clauses *P* and *Q* should be produced as a single integrated intonation unit without a prosodic boundary, as they form a unified semantic unit. Previous studies by Couper-Kuhlen (1996) and Günthner (1996) examined this proposition and observed that subjective causality was uttered as two intonation units with an F0 reset at the beginning of the second unit, whereas objective causality was uttered as a single intonation unit with an integrate F0 contour. These findings appeared to support the hypothesis put forth by Rutherford (1970) and Sweetser (1990). However, these findings were based on auditory and visual inspection of F0 trajectories and lacked statistical analyses, making it difficult to generalize the results across speakers and utterances. In contrast, den Ouden (2004) employed a statistical approach to compare the prosodic realizations of subjective and objective causality in read speech. The researchers found that the pause preceding the second clause was longer in subjective causality than in objective causality. As longer pauses can contribute to the perception of stronger prosodic boundaries (Cole, 2015; Wagner & Watson, 2010), this finding seems to support the proposition by Rutherford (1970) and Sweetser (1990). However, one potential issue with this study is that the subjective and objective causality being compared did not have the same causality order. Specifically, the subjective causality used in the study involves forward causality (ARGUMENT-CLAIM/CONCLUSION), whereas the objective causality involves backward causality (CONSEQUENCE-CAUSE). This disparity in causality order raises the question of whether it could have caused the difference in the duration of the pause and thus casts a doubt on the study’s conclusion.

Directly relevant to this topic are studies on the prosodic realizations of stance, an umbrella term for subjectivity, evaluation, opinion, and assessment (Ward et al., 2018). These studies have demonstrated that words or simple sentences conveying opinions had a wider F0 range, fewer pauses, faster speaking rates, and longer stressed vowels compared to those presenting factual information (Freeman, 2014, 2019; Morency et al., 2011). Considering that complex sentences expressing subjective causality involve subjectivity, it is conceivable that such sentences might exhibit similar prosodic features identified in these aforementioned studies.

This study aimed to thoroughly examine the role of prosody in conveying subjective and objective causality in the absence of morpho-syntactic markers. English was selected as the target language due to the common usage of a general causal connective for expressing these two types of causality. Two production experiments were conducted to examine the prosodic realization of subjective and objective causality, with Experiment 1 focusing on backward order and Experiment 2 on forward order. In these two experiments, a newly designed dialogue task was employed to create naturalistic conversation settings where participants expressed subjective and objective causality. A wide range of prosodic features was examined, including not only static acoustic-phonetic measurements such as F0 maximum, F0 minimum, speech rate, and duration, but also the dynamic characteristics of F0 trajectories derived using Functional Principal Component Analysis (FPCA), as described by Gubian et al. (2015).

Our working hypothesis posits that prosody plays a role in this context, aligning with the proposition put forth in previous discourse studies. Furthermore, this hypothesis finds support in existing evidence illustrating the active involvement of prosody in conveying various linguistic meanings in the absence of lexical markers. For instance, previous studies have demonstrated that questions lacking morpho-syntactic marking often exhibit prosodic features such as a final rise (Haan, 2001). This phenomenon extends to other linguistic contexts as well, including the marking of word boundaries (Mattys & Melhorn, 2007), the differentiation between questions and statements (Petrone & Niebuhr, 2014; van Heuven, 2017), the disambiguation of syntactic ambiguity (Snedeker & Trueswell, 2003), the marking of information structure (Braun & Chen, 2010), the distinction of various functions of discourse markers (see Hirschberg et al. (2020) and Cole (2015) for extensive reviews), and the expression of higher levels of linguistic meanings such as sentiment (Morency et al., 2011), commitment (Prieto & Roseano, 2021), and sarcasm (Afflerbach, 2015; Chen & Boves, 2018).

Regarding the specific role of prosody in expressing subjective and objective causality, we formulated the following predictions. First, we predict that subjective causality will be produced with a wider F0 range or faster speaking rate than objective causality, because it involves opinions. To test this prediction, we analyzed the F0 maximum and F0 minimum in both the first and second clauses, as well as the speaking rate in each clause.

In addition to the static F0 measurements, we also examined the overall F0 contour shape. Previous studies have revealed that the degree of “matter-of-fact” conveyed in an utterance decreases as the F0 peak position of the accented word in an utterance moves from early to medial to late F0 synchronization. Early peaks convey the highest degree of “matter-of-fact,” whereas late peaks indicate speakers’ evaluation (Kohler, 2005). Furthermore, an attitudinal meaning contrast is reflected in the contrast between utterance-final rise and fall, with utterance-final F0 rises more strongly inviting the conversation partner to participate in the conversation compared to utterance-final F0 falls (Kohler, 2004). These findings suggest that utterances conveying subjective causality may exhibit a later F0 peak and an F0 final rise in comparison with utterances expressing objective causality. To test this prediction, we employed FPCA (Gubian et al., 2015) to obtain measurements that describe the shape of F0 contours.

Third, considering the argument in discourse literature that the meanings of *P* and *Q* are more distantly related in subjective causality than in objective causality (Sweetser, 1990; Verhagen, 2005; Zufferey, 2012), we predict that utterances expressing subjective causality may exhibit less integrated prosody or larger between-utterance prosodic boundary in comparison with utterances expressing objective causality. Specifically, we examine whether there is a difference in the duration of the pause (the silent interval) preceding the causal connective or the duration of the connective itself between subjective and objective causality. The pause duration will be longer in subjective causality compared with objective causality, as a longer pause serves as a strong cue for a larger prosodic boundary (Cole, 2015; Wagner & Watson, 2010).

## 2 Experiment 1: backward causality

### 2.1 Participants

Fifteen native speakers of American English (11 females, 4 males) between the ages of 22 and 25 (mean age: 23 years) participated in the experiment. The participants were recruited from Utrecht University through the university’s international office and the first author’s social networks. All participants reported normal hearing and speaking abilities and no experience in acting. A payment of €10 was provided upon completion of the experiment.

### 2.2 Task

The experiment involved a dialogue task that was designed to elicit utterances expressing either subjective or objective causality in a naturalistic conversational setting while maintaining uniformity in the participants’ responses. During the task, the participants engaged in mini-conversations with an interlocutor based on information presented on PowerPoint slides. They were instructed to construct new sentences using short sentences shown on the slides and producing them as responses to the interlocutor’s questions.

**Figure 1.**
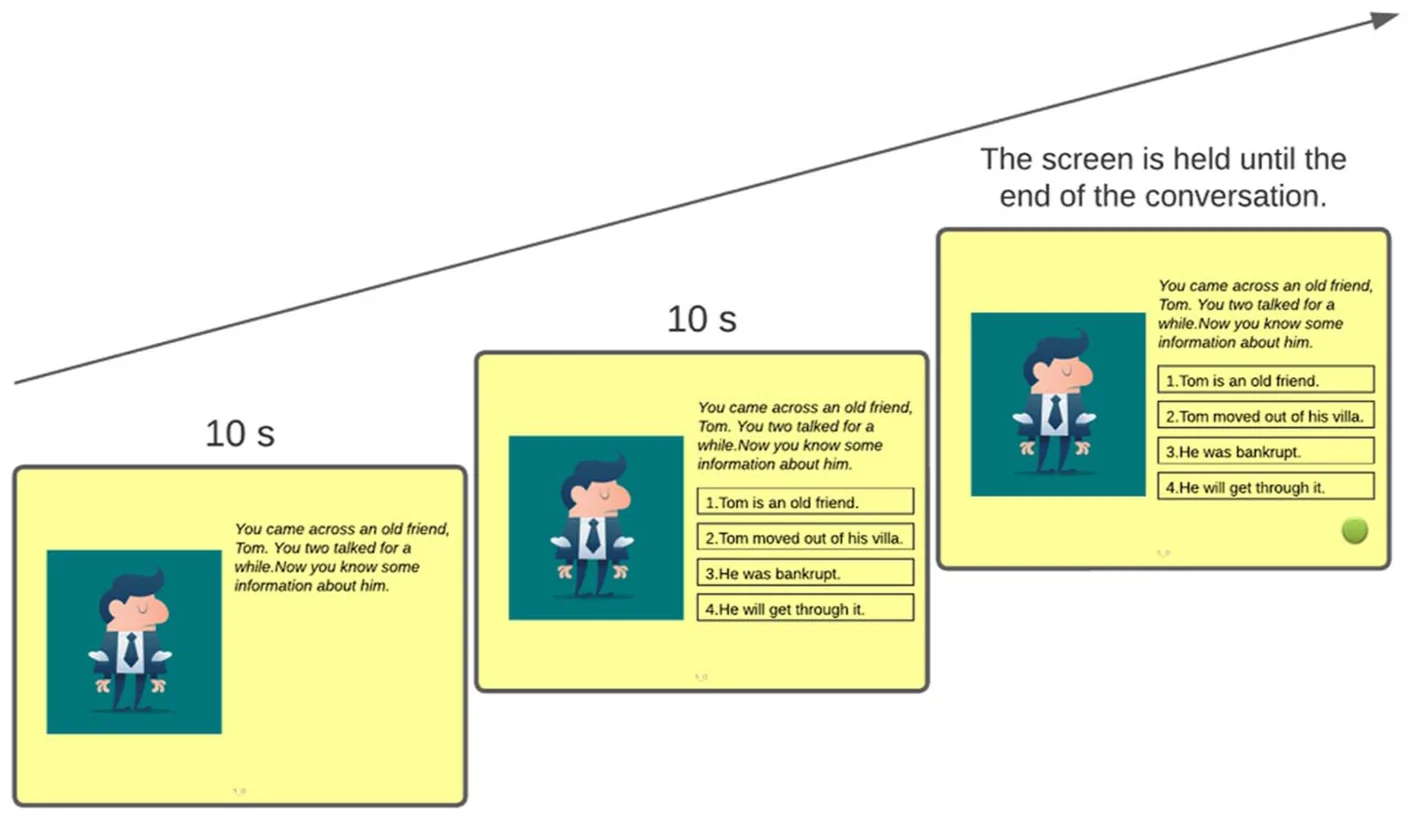
The timeline of an experimental trial consisted of three slides (for the objective item “*Tom moved out of his villa because he was bankrupt*”).

The experimental trials were structured as follows (refer to Figure 1 for the timeline). First, the participants saw a background story (texts in italics in Figure 1) and an accompanying image, which set up the conversational context and primed the participants for the intended causality (Scholman & Demberg, 2017). The background stories varied by the condition: in the objective causality condition, the participants were told they were in direct contact (e.g., chatting, as shown in Figure 1) with an imaginary acquaintance and relaying to him or her an event that occurred in reality, as described in the following slide. Conversely, in the subjective causality condition, the participants were encouraged to view the upcoming information on the following slide as their own opinions or evaluations of the character or event mentioned on the slide (e.g., “*You have the following impression about him*”). After 10 s, four short sentences appeared simultaneously on the slide, with a causal (or concessive) relationship between the second and third sentences (see the next section for details). After another 10 s, a green button appeared in the bottom-right corner, signaling the start of the dialogue with the experimenter. This trial structure ensured that the elicited target utterances conveyed the intended type of causality. It also ensured that the participants read all the information displayed on the slides without skipping any content. In addition, by allowing ample time for the participants to process the discourse link between the sentences, especially between Sentences 2 and 3, this structure could reduce the participants’ cognitive pressure when conceptualizing subjective causality, potentially mitigating any physiological effects on prosody (Paulmann et al., 2016).

The interlocutor posed three questions or requests per slide, and the participants received instructions before the experiment on how to respond using the sentences on the slides (see the Supplementary Material). The first question initiated the conversation, such as “*Who is Tom?*” for the trial shown in Figure 1. To answer this question, the participants were told to use the first sentence on the slide. The interlocutor’s second utterance elicited a target response expressing either subjective or objective causality (or a filler utterance expressing a concessive relation). In the target condition, the second utterance was “*Tell me something about X*” (or a question with a similar meaning). The utterances that elicited filler items varied based on the context. To respond to the second utterance, the participants were instructed to combine the second and third sentences on the slide into one sentence using either “because” or “however” without changing the order of the two sentences. For example, for the slide in Figure 1, the expected response to the second question was “*Tom moved out of his villa because he was bankrupt.*” The third question ended the conversation, and its answer was the last sentence on the slide. It is important to note that the participants only need to record the four short sentences on the second slide, not the background stories. Those background stories were only accessible to the participants, and not to the interlocutor, as the interlocutor could not see the participants’ screen during the task.

During the task, the participants independently processed the relationship between Sentences 2 and 3 and chose the connective they saw fit. They received no feedback from the experimenter regarding the correctness of their answer. They were strongly encouraged to treat the sentences as their own words and to produce them as naturally as they would in a real conversation, rather than reading them aloud.

### 2.3 Materials

The target sentences were the responses to the second question in each conversation. These target sentences were designed to express either subjective or objective causality. Each speaker produced a total of 30 such sentences, all conveying backward causality using the connective “because.” Some of these sentences were taken from previous studies on subjectivity in causality (Canestrelli et al., 2013; Rodríguez-Gómez et al., 2016; Sanders & Evers-Vermeul, 2019; Traxler et al., 1997), whereas the remaining ones were newly composed.

The 30 target sentences formed 15 pairs, with each pair consisting of one objective causality sentence (see Sentence [5]) and one subjective causality sentence (see Sentence [6]; see Supplementary Materials for a complete list of sentences). The sentences were carefully selected to ensure comparability in terms of length and syntactic complexity.

[5]  [Tom moved out of his villa]_C1_ [because he was bankrupt]_C2_.

[6]  [Tom knew nothing about business]_C1_ [because he was bankrupt]_C2_.

For the two sentences (hereafter “items”) in each pair, the subordinate “because”-clauses (i.e., the second clauses, denoted by “C2” in subscripts in Sentences [5] and [6]) were identical, introducing the same real-world event (e.g., “*He [Tom] was bankrupt*” in Sentences [5] and [6]), whereas the main clauses (the first clauses, denoted by “C1” in Sentences [5] and [6]) were different. The main clause stated the actual consequence of the event stated in C2 in the objective condition (e.g., “*Tom moved out of his villa*” in Sentence [5]), but an opinion or conclusion regarding the event stated in C2 in the subjective condition (e.g., “*Tom knew nothing about business*” in Sentence [6]).

These items underwent proofreading by three native speakers of American English before being included in the task. Furthermore, the authors verified the conveyed causal relation in each item using a paraphrase test with sentence frames (Pander Maat & Degand, 2001; Sanders, 1997). The sentence frame used to test objective causality was “*The fact that [P] led to the consequence that [Q]*,” whereas the one used for subjective causality was “*The fact that [P] leads to one’s conclusion (claim or advice) that [Q].*” An item was considered valid if its paraphrase had the same meaning as the original sentence. All items passed the paraphrase test.

Subsequently, the target items were divided into two lists, with each list containing approximately the same number of items for the two causal categories, but only one item from each pair. List 1 consisted of seven subjective items (from Pairs 1–7) and eight objective items (from Pairs 8–15), whereas List 2 comprised eight subjective items (from Pairs 8–15) and seven objective items (from Pairs 1–7). To conceal the purpose of the study, 10 filler items conveying concessive relations involving “however” were added to each list.

### 2.4 Procedure

The participants were individually tested in a sound-treated booth located in the phonetics lab at the Institute for Language Sciences at Utrecht University.

Upon the arrival of each participant, the experimenter (the first author) informed them, without revealing the actual research goal, that the experiment aimed to collect natural speech samples for teaching purposes. The experimenter then introduced the participant to the interlocutor, a female native speaker of American English, instructing the participant to interact with her as they would with a friend in real-life conversations. Afterward, the experimenter invited the participant to enter a sound booth and sit with the interlocutor at a table, where a recorder and a computer monitor were positioned facing the participant’s direction.

Once seated, the participant received an oral explanation of the dialogue task, the slide design, and the instructions from the interlocutor. Then, they underwent two practice trials. After completing the practice trials, the participant proceeded to perform trials on Lists 1 and 2 (or vice versa), with a 5-min break in between. The items on each list were presented in a random order, and the participants clicked the mouse to proceed after each trial. The conversations were recorded in a PCM (.WAV) audio format using a ZOOM 1 portable digital recorder (sampling rate of 44.1 kHz, 16 bit, stereo). The entire experiment lasted approximately 1 hour.

### 2.5 Data annotation and measurements

The recordings obtained from the dialogue task were processed using *Praat* (Boersma & Weenink, 2018) with the following steps. First, the onset and offset of the target utterance, that is, the utterance elicited by the second question, were marked in each dialogue. Subsequently, the target utterances were extracted, resulting in a total of 450 utterances, with 4 being discarded due to disfluencies. In the third step, the boundaries of the first clause, second clause, and connective were annotated. For utterance-initial words beginning with plosives, the boundary was set at the burst of the plosives. Fourth, the F0 contour of each utterance was inspected and octave jumps were manually corrected. Fifth, several prosodic measurements were extracted using a Praat script, including the F0 maximum and F0 minimum (in semitones [st] relative to 1 Hz) in each clause, the duration of the connective (in seconds [s]), and the duration of the silent interval preceding the connective. In addition, the speech rate in each clause, defined as the number of syllables produced per second, was calculated (Hardcastle et al., 2010).

In addition to the aforementioned static measurements, we used FPCA (Gubian et al., 2015) to obtain measurements describing the shape of F0 contours. FPCA offers advantages over phonological frameworks such as Tones and Break Indices (ToBI; Beckman et al., 2005), as it can better handle the variability of F0 contours. In brief, FPCA represents each F0 contour as a mathematical function comprising the average contour and several principal component curves (FPCs). Each FPC is associated with a weight that determines its contribution to approximating the original F0 contour. In our study, FPCA was used to model the F0 contours of the first clause and the connective separately, following the procedure outlined in Gubian et al. (2015).

First, the original F0 contours were smoothed through spline interpolation and linearly normalized for duration. For the connective “because,” the smoothed contours were additionally scaled based on common “landmarks,” specifically the boundary between the two syllables. Subsequently, functional PCA was conducted to extract FPCs and calculate the weights for each curve.

**Figure 2.**
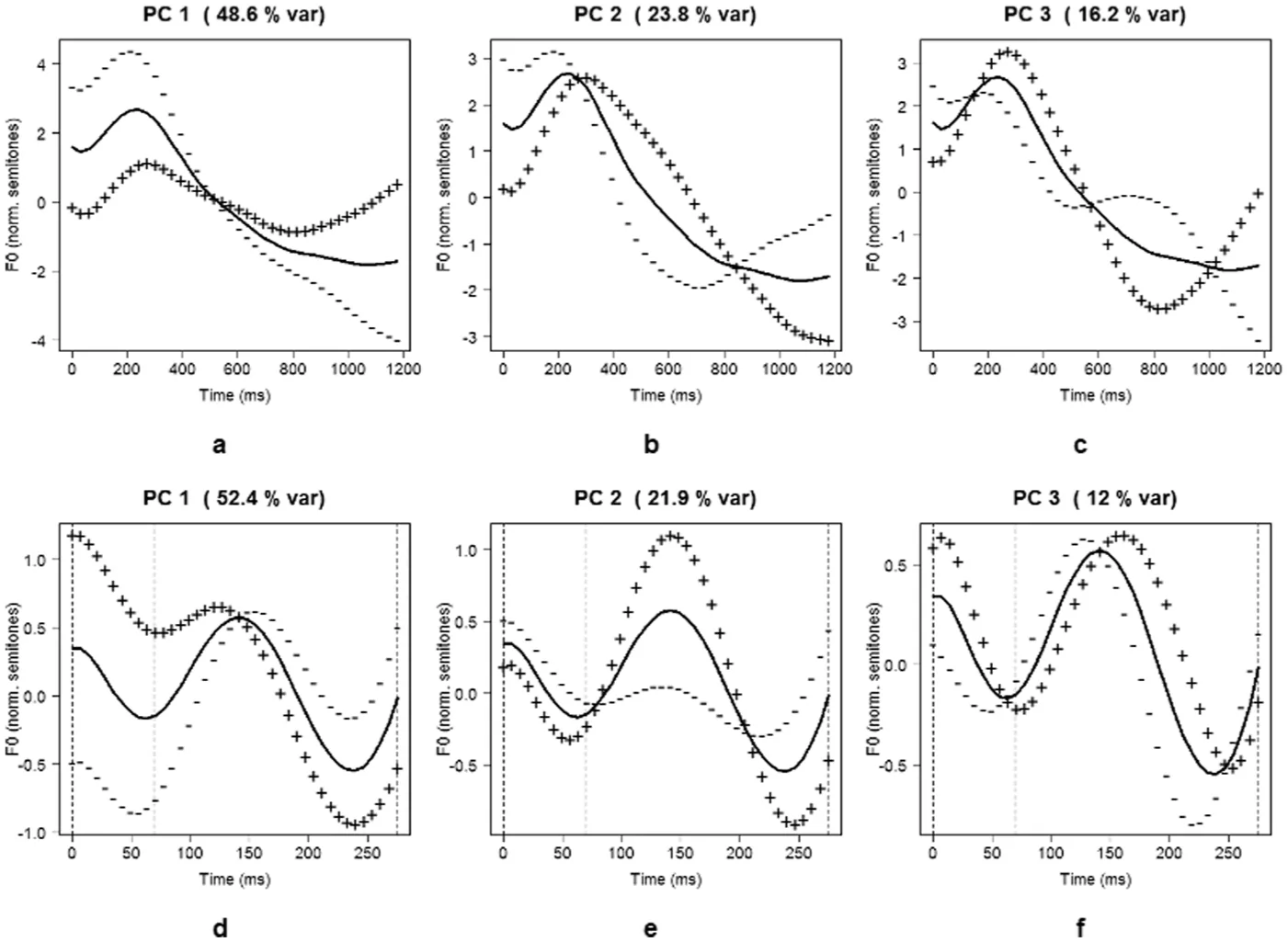
The effect of each Functional Principal Component (FPC) on the mean F0 contour is depicted for both the first clause (panels a–c) and the connective (panels d–f). From left to right: FPC1, FPC2, and FPC3. *Note*. Each panel displays the mean F0 contour (normalized F0 in semitones over time in milliseconds) as a solid line. The “+/-” lines illustrate the outcome curves after adding or subtracting one standard deviation of an FPC from the mean F0 curve, respectively. In the bottom panel, vertical dotted lines indicate syllable boundaries within the word “because.” The x-axis represents time in milliseconds, and the y-axis represents frequency values in semitones.

Figure 2 demonstrates the effect of each FPC on the mean F0 contour (top row: the main clause; bottom row: the connective). In each panel, the solid line corresponds to the mean F0 contour, whereas the “+” and “-” curves indicate the F0 contours after adding or subtracting one standard deviation of an FPC to or from the average contour, respectively. The vertical dash line in the bottom panel indicates the boundary between the two syllables in “because.” As can be seen in Figure 2(a) and (d), FPC1 affects the overall linear slope and the degree of final rises of the mean F0 contour. A positive weight of FPC1 flattens the contour’s slope for the first clause and also increases the degree of the final rise, whereas a negative weight enhances its steepness. Conversely, for the connective, the effects of positive and negative weights of FPC1 are reversed. FPC2 primarily modifies the curvature of the mean contour. In the case of the first clause (Figure 2(b)), a positive weight of FPC2 shifts the onset of the F0 fall toward the right end, resulting in a less steep fall. Conversely, a negative weight of FPC2 shifts the onset of the fall toward the left end, leading to a steeper fall. Regarding the F0 contour of the connective “because” (Figure 2(e)), a positive weight of FPC2 shifts the onset of the F0 rise toward the left end, making the middle of the contour more convex. Conversely, a negative weight of FPC2 shifts the onset of the rise toward the right end, resulting in a less convex middle portion. FPC3 adjusts the temporal alignment of peaks and valleys in both cases, with a positive weight delaying the occurrence of a peak or valley and a negative weight advancing it in time (see Figure 2(c) and (d)).

A total of 14 measurements were obtained, and the complete list can be found in Table 2.

**Table 2.** Measurements Used in Experiment 1.

Measurements	Descriptions
connective_Dur	The duration of the connective “because”
pause_Dur	The duration of the silent interval between the first clause (C1) and the second clause (C2)
connective_PC1, _PC2, _PC3	The weights of the first three FPCs for the F0 contour of the connective
f0_Max_C1, f0_Max_C2	The maximum F0 values in C1 and C2
f0_Min_C1, f0_Min_C2	The minimum F0 values in C1 and C2
speechR_C1, speechR_C2	The speech rate in C1 and C2, respectively
C1_PC1, _PC2, _PC3	The weights of the first three FPCs for the F0 contour of C1

### 2.6 Statistical analysis

The effect of subjectivity on each measurement was assessed using Bayesian statistics in R (version 3.6.3, R Core Team, 2020) with the brms package (Bürkner, 2017), which serves as a wrapper package for the probabilistic programming language Stan (Carpenter et al., 2017). We opted for a Bayesian approach over the frequentist approach because it allows us to directly assess the probability of a hypothesis given the data, which aligns with our research objective. In contrast, the frequentist approach focuses on the probability of observing the data given the hypothesis. To illustrate the distinction between the two types of probability, consider the difference between the probability of having Covid-19 given coughing (relatively low) and the probability of coughing given having Covid-19 (relatively high).

We fitted a series of Bayesian regression models for each acoustic measurement presented in Table 2 (code and data are available at https://osf.io/z2k7r/). The predictor variables included subjectivity, which indicated the type of causality and was contrast-coded as +0.5 for subjective causality and −0.5 for objective causality. In addition, we included gender as a predictor, as it is known to have effects on several of our acoustic-phonetic measurements (Cole, 2015). Gender was contrast-coded as +0.5 for males and −0.5 for females. We also examined the interaction between subjectivity and gender. The grouping variables in the models were subject and item.

These effects were incrementally added to the models, beginning with an intercept model (m0) that included only the grouping variables subject and item. Subsequently, more complex models were constructed to include the constant effects and the interaction term (in the order of gender, subjectivity, and gender: subjectivity). To account for the variability across individuals and items, models with random slopes for subjectivity were developed, considering both subject-specific and item-specific variations.

The contribution of each term to model fit was assessed using Bayes factors. Bayes factors serve as a measure similar to the likelihood ratio between two models and are commonly used to evaluate the presence of the effect of interest in the data. The Bayes factors are computed by the bayes_factor(m1, m0) function in the brms package, where m1 includes the effect of interest and m0 does not. The resulting Bayes factors, denoted as *BF_10_* (with subscripts 1 and 0 representing m1 and m0, respectively), quantify the extent to which the evidence supports m1 over m0 (Lee & Wagenmakers, 2014; Nicenboim & Vasishth, 2016). A larger *BF_10_* value indicates stronger support for m1. Bayes factors on a natural scale are straightforward to interpret as they indicate how much more likely m1 is compared to m0. Thus, the interpretation of Bayes factors in the main text adheres to their natural scale, using the criteria outlined by Lee and Wagenmakers (2014) following Jeffreys (1998). However, in figures, we adopted a logarithmic scale, which has the advantages of setting zero as the point of indifference between the two models being compared (corresponding to a Bayes factor of 1 on the natural scale), and making equal increments correspond to equal changes in the relative probabilities. The correspondence between Bayes factors on a natural scale and a logarithmic scale is presented in Table 3.

**Table 3.** Interpretation Scheme for Bayes Factors on a Natural Scale and a Logarithmic Scale, According to Raftery (1995).

Interpretation	*BF_10_*	log (*BF_10_*)
Very strong support for m0	< 0.0067	< −5
Strong support for m0	0.0067 to 0.05	−5 to −3
Positive support for m0	0.05 to 0.33	−3 to −1
Weak support for m0	0.33 to 1	−1 to 0
No support for either model	1	0
Weak support for m1	1 to 3	0 to 1
Positive support for m1	3 to 20	1 to 3
Strong support for m1	20 to 150	3 to 5
Very strong support for m1	> 150	> 5

It is important to acknowledge that Bayes factors can be influenced by the prior assumptions incorporated in the models, which represent the beliefs held by the model before observing the data. Consequently, interpreting Bayes factors can be challenging. To address this issue, we followed the recommended practice of computing Bayes factors under different priors (Nicenboim et al., 2020; Vasishth et al., 2018). In our study, we evaluated each effect using three different priors, all of which were normal distributions—normal (0, *SD* ) with increasingly larger standard deviations (*SD* ). The selection of appropriate hyperparameters for the priors was guided by previous research on the prosodic correlates of discourse structure (den Ouden et al., 2009). The computation of Bayes factors was performed using bridge sampling with four chains and 10,000 iterations, with a warm-up phase of 2,000 iterations. One might argue that, with the numerous acoustic measurements and multiple factors under investigation, the reported Bayes factors might suffer from multiple-testing issues, increasing the risk of Type I errors. In general, however, Bayesian analyses do not suffer from this problem, provided that all relevant comparisons are included in the analyses for each measurement (Dienes, 2016; Grossman, 2011), as is the case here. Nevertheless, caution in interpreting the results is required because of the possible interrelations between the acoustic-phonetic variables under study.

In the following result section, we first presented the Bayes factors for each effect on each acoustic measurement. In the interest of space, we concentrated on the acoustic measurements where there is evidence supporting the effect of interest in the main text, specifically when Bayes factors approach or exceed a value of 3. We provided model estimates in the form of a posterior probability distribution, represented by the mean and a 95% credible interval (95% CrI). The 95% CrI is an equal-tailed interval that represents a range of values within which a parameter is considered to lie with 95% probability. Unless stated otherwise, the posterior distributions mentioned in the main texts were estimated using the model with the prior having the largest *SD* (i.e., the least informative prior). For effects lacking support from Bayes factors, corresponding model estimates can be found in the Supplementary Materials.

### 2.7 Results

**Figure 3.**
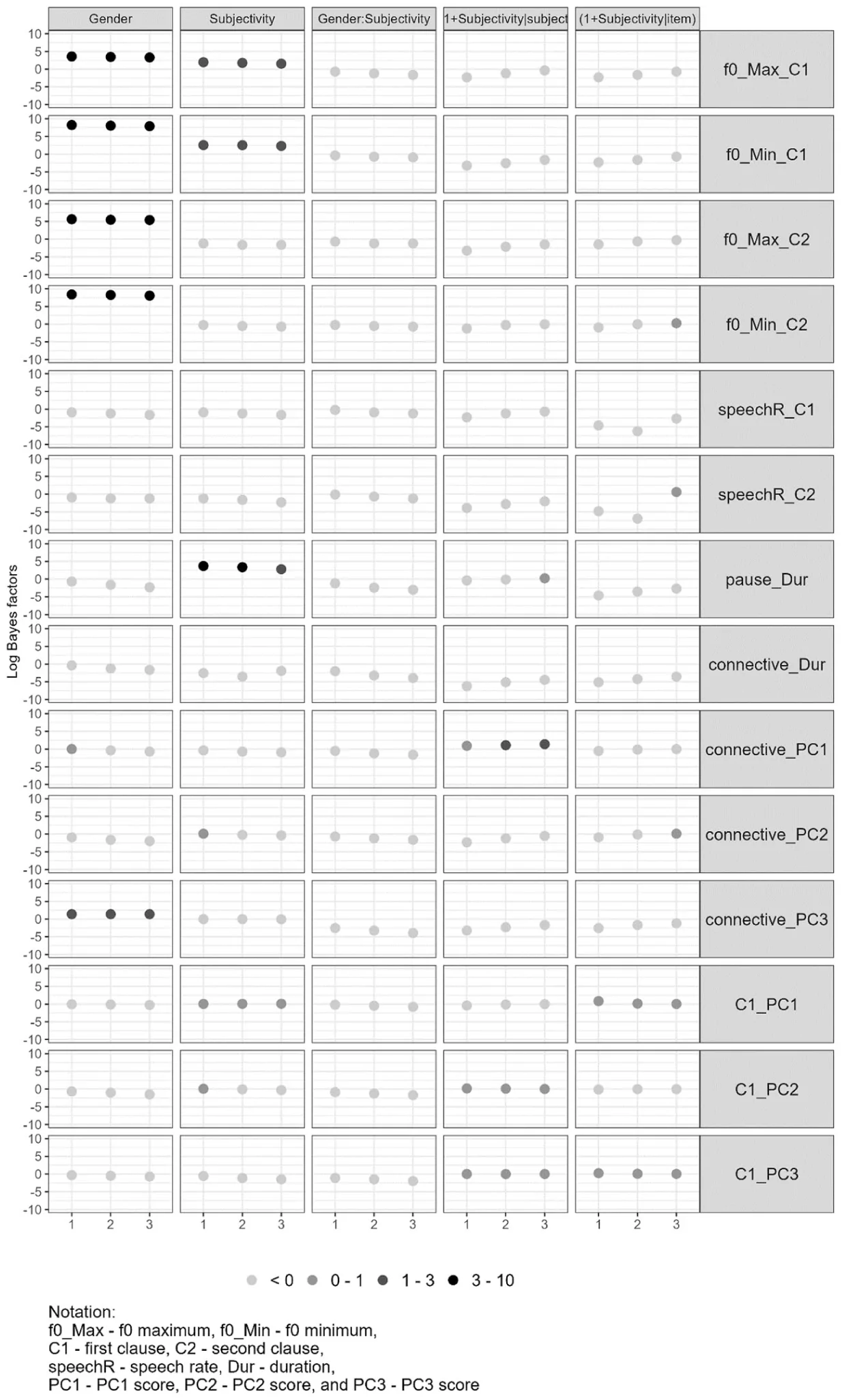
Bayes factors (plotted on a logarithmic scale) evaluating the impact of each factor (columns) on each acoustic measurement (rows). *Note*. The factors include gender, subjectivity, the interaction between gender and subjectivity (gender:subjectivity), the random slopes of subjectivity by subject (1 + subjectivity|subject) and by item (1 + subjectivity|item). The notation key is provided below the plot. The values of Bayes factors are presented as dots, color-coded to indicate their value ranges: black for values larger than 3, dark gray for values between 1 and 3, mid-gray for values between 0 and 1, and light gray for values < 0.

Figure 3 presents the Bayes factors on a logarithmic scale, evaluating the impact of each effect on each acoustic measurement, calculated three times under different prior assumptions. The Bayes factors are color-coded to indicate their value ranges.

The results showed evidence of subjectivity affecting the F0 maximum and minimum in the first clause, and the duration of the pause before the connective, with varying degrees of evidence strength. Concerning these measurements, there was little support for models incorporating a random slope of subjectivity by speaker or by item, suggesting little variability among speakers or items concerning the effect of subjectivity. Regarding the PC1 score of the F0 contour of the first clause, the Bayes factors evaluating the effect of subjectivity ranged from 1.11 to 1.05, indicating that the models with and without this effect had similar likelihood. There was no discernible evidence of subjectivity affecting the remaining measurements. Next, we focused on the measurements that showed an effect.

First, there was strong evidence demonstrating the influence of subjectivity on the F0 minimum in the first clause and the pause duration before the connective, as indicated by Bayes factors larger than 10. In terms of the F0 minimum in the first clause, the effect of subjectivity had a posterior distribution with a central tendency at −0.69 st, with a 95% CrI of [−1.15 st, −0.23 st]. This finding suggested that the F0 minimum in the first clause was lower in subjective causality than in objective causality. With respect to the duration of the pause before the connective, the effect of subjectivity was estimated with a mean of 0.05 s and a 95% CrI of [0.02 s, 0.08 s]. The estimate revealed that the pause duration preceding the connective was 50 ms longer in instances of subjective causality than in cases of objective causality.

In addition, there was moderate evidence indicating subjectivity’s impact on the F0 maximum in the first clause, as Bayes factors for this is between 3 and 10. This effect was predicted with the mean at 0.59 st and a 95% CrI [0.14 st, 1.04 st]. It suggested that the F0 maximum in the first clause was higher in subjective causality than in objective causality.

One reviewer pointed out that in 7 out of the 15 subjective experimental items, part of the target sentence had already been presented to the participants as background information; the pragmatic status as “given” information might then have affected the prosody of the target sentence. However, the participants needed to present this (presumably given) information in their spoken target sentence as “new” information to the interlocutor, consistent with the task demands in both the remaining subjective causality items and the objective causality items. Moreover, a post hoc re-analysis including “givenness” as an additional factor (see Supplementary Materials at https://osf.io/z2k7r/ for details and results) showed no evidence that “givenness” influenced any of the acoustic measurements.

### 2.8 Interim summary

The findings from Experiment 1 provided clear evidence of distinct prosodic realizations between subjective and objective causality in backward order. Specifically, we observed a longer pause preceding the connective in subjective causality as compared with the connective in objective causality. In addition, we found that subjective causality exhibited a “higher F0 maximum” and a “lower F0 minimum,” resulting in an “expanded F0 range,” than objective causality.

Although several prosodic differences were identified between subjective and objective causality in the backward order, it remains unclear whether these findings extend to subjective and objective causality in the forward order, where *P* precedes *Q*. This question is addressed in Experiment 2.

## 3 Experiment 2: forward causality

### 3.1 Participants

Experiment 2 involved a total of 15 participants (11 females, 4 males; age range: 22–26 years; mean age: 24 years), all of whom were native speakers of American English. The experiment was conducted approximately 6 months after Experiment 1. The participants were recruited from Utrecht University through the university’s international office, as well as through the first author’s social networks. Three of the participants had previously participated in Experiment 1. However, they were unaware of the true research objective and reported no recollection of the utterances used in Experiment 1. All participants confirmed that they had no prior acting experience.

### 3.2 Task and procedure

The participants were tested in a sound-treated booth located in the phonetics lab at the Institute for Language Sciences at Utrecht University. They completed the same dialogue task as conducted in Experiment 1, following the same procedure detailed in Sections 2.2 and 2.4. Similar to Experiment 1, PowerPoint slides were employed to present the information during the task. The slide design replicated that of Experiment 1, with the exception that the positions of Sentences 2 and 3 were reversed on each slide. This modification resulted in a change in the causality order between the two sentences, shifting from a backward order to a forward order. The instructions provided to the participants were identical to those given in Experiment 1, with the exception that the two optional connectives were changed to “so” and “but.” The participants received a participation fee of 10 euros.

### 3.3 Materials

The target utterances elicited from the task were modified versions of the items used in Experiment 1, all conveying forward causality with the causal connective “so.” Similar to Experiment 1, there were 15 pairs of such items in this experiment, with each pair comprising one sentence expressing subjective causality and one sentence expressing objective causality. In each pair, the first clauses (labeled as “C1” in the subscripts in Sentences [7] and [8]) remained the same, introducing a real-world event (e.g., “*Tom was bankrupt*” in Sentences [7] and [8]), whereas the second clauses (labeled as “C2” in the superscripts) were different. The second clause in the objective condition stated the consequence of the event described in C1 (e.g., “*He moved out of his villa*” in Sentence [7]), whereas the second clause in the subjective condition expressed an opinion regarding that event (e.g., “*He knew nothing about business*” in Sentence [8]).

[7]  [Tom was bankrupt]_C1_ [so he moved out of his villa]_C2_.

[8]  [Tom was bankrupt]_C1_ [so he knew nothing about business]_C2_.

Furthermore, the current experiment utilized the filler items from Experiment 1 to disguise the actual research goal.

### 3.4 Data annotation and statistical analysis

**Figure 4.**
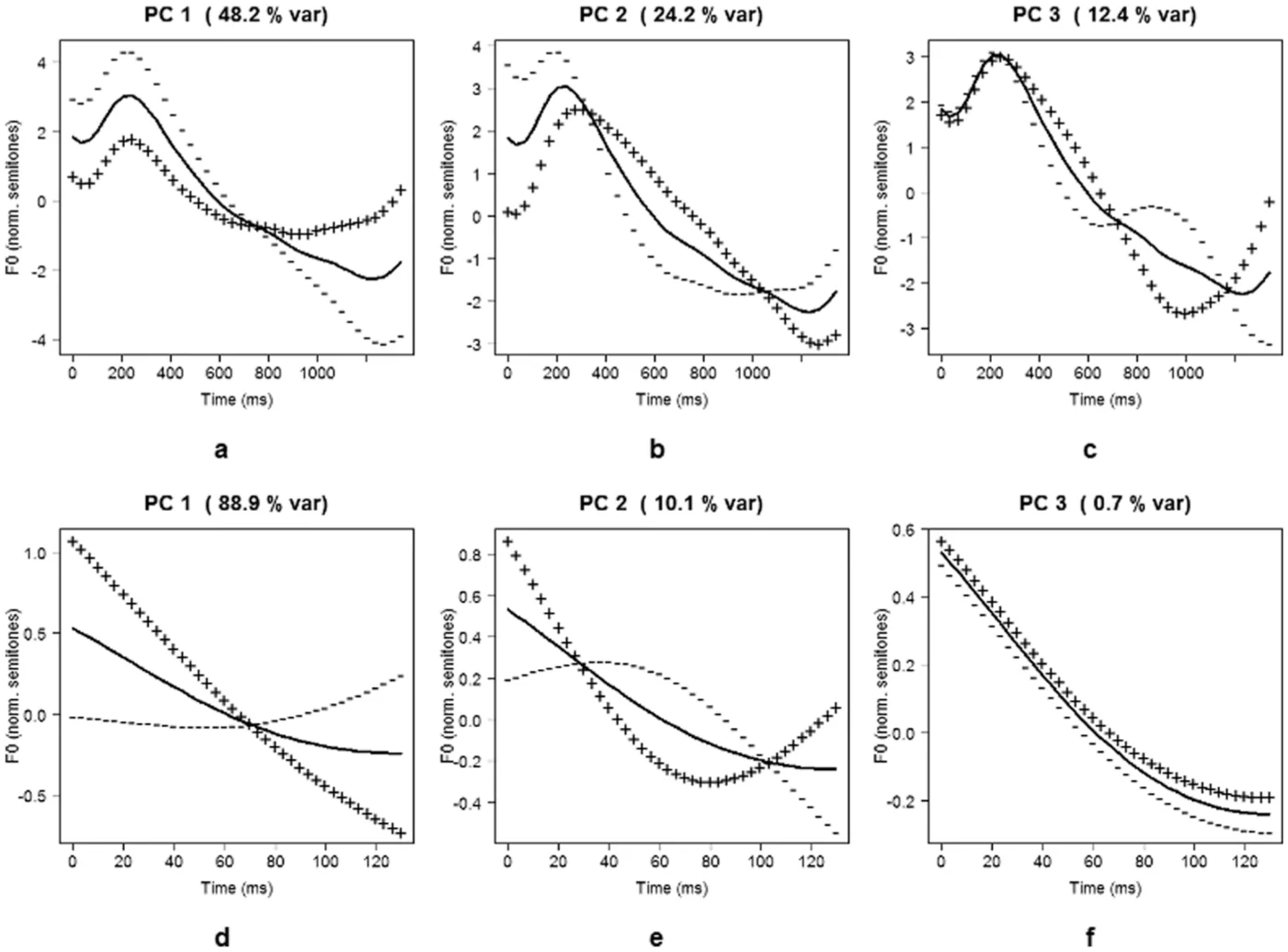
The effects of FPCs on the mean F0 contour of the first clause (panels a–c) and the connective (panels d–f). From left to right: FPC1, FPC2, and FPC3. *Note*. In each panel, the solid line represents the mean F0 contour (normalized F0 in semitones over time in ms), the “+” line illustrates the outcome curve when one standard deviation of the FPC is added to the mean F0 curve, and the “-” line represents the outcome curve when one standard deviation of the FPC is subtracted from the mean F0 curve. The x-axis denotes time in milliseconds, and the y-axis represents F0 values in semitones.

The recordings obtained from the experiment underwent the same processing steps as in Experiment 1 (see Section 2.5 for detailed information). In addition, we employed FPCA to model the F0 contours of the first clause and the connective “so.” In Figure 4, the effects of the first three FPCs on the mean F0 contour of the first clause are depicted in the top panel, whereas the bottom panel showcases the effects on the mean F0 contour of the connective “so.”

The top panel of Figure 4 demonstrates that the effects of the three FPCs on the mean F0 contour of the first clause were the same as those on the mean F0 contour of the first clause in Experiment 1 (see Section 2.5 for detailed descriptions).

Regarding the effects of the three FPCs on the F0 contour of the connective “so,” Figure 4(d) illustrates that FPC1 affects the overall linear slope of the mean F0 contour. A positive weight assigned to FPC1 results in a steeper slope, whereas a negative weight leads to a flatter slope. FPC2 alters the curvature of the mean curve, with a positive weight increasing concavity and a negative weight increasing convexity (Figure 4(e)). FPC3 primarily modulates the intercept of the curve with the y-axis, where a positive weight yields a larger intercept and a negative weight yields a smaller intercept (Figure 4(f)).

The measurements and the statistical method employed in this experiment were identical to those employed in Experiment 1 (see Sections 2.6 for detailed information).

### 3.5 Results

**Figure 5.**
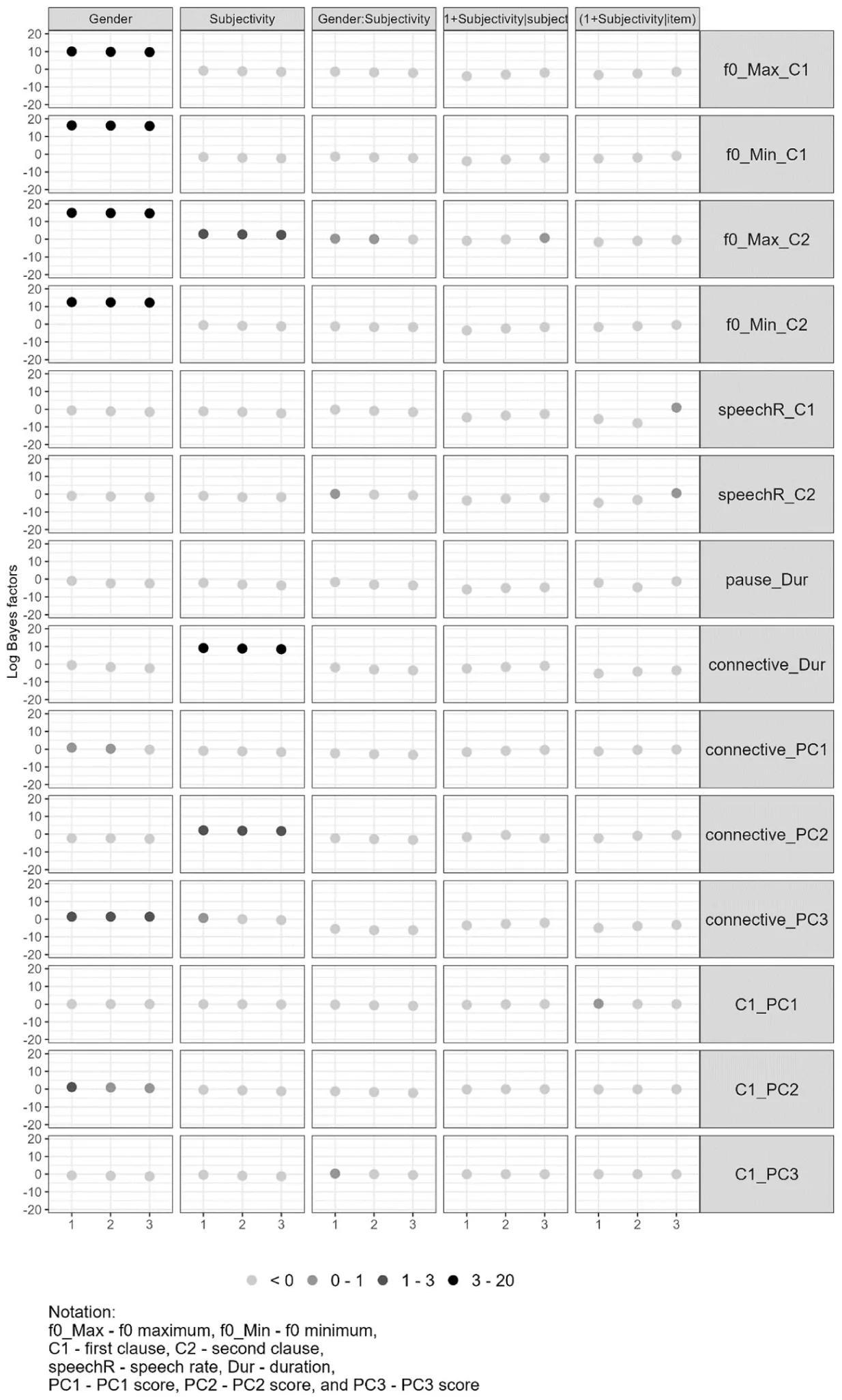
Bayes factors (plotted on a logarithmic scale) evaluating the impact of each factor (columns) on each acoustic measurement (rows). *Note*. The factors include gender, subjectivity, the interaction between gender and subjectivity (gender:subjectivity), the random slopes of subjectivity by subject (1 + subjectivity|subject) and by item (1 + subjectivity|item). The notation key is provided below the plot. The values of Bayes factors are presented as dots, color-coded to indicate their value ranges: black (values > 3), dark gray (values between 1 and 3), mid-gray (values between 0 and 1), and light gray (values < 0).

Figure 5 presents the Bayes factors on a logarithmic scale, assessing the impact of each effect on each acoustic measurement. As in Experiment 1, the calculations were performed three times using different prior assumptions. To aid interpretation, the Bayes factors are color-coded to represent their value ranges.

The results indicated that subjectivity had an impact on the F0 maximum in the second clause, the duration of the connective, and the PC2 score of the F0 contour of the connective. However, for the other measurements, the Bayes factors evaluating the impact of subjectivity were very low, indicating the absence of any discernible effect on these acoustic measurements. In what follows, we focused on the three measurements that demonstrated an impact of subjectivity.

First, the Bayes factors assessing the effect of subjectivity on the F0 maximum in the second clause all exceeded 10, providing strong evidence for the effect. The estimated mean was 0.72 st, with a 95% CrI of [0.28 st, 1.16 st]. This implied that the F0 maximum in the second clause was 0.72 st higher in cases involving subjective causality compared with those involving objective causality.

Furthermore, the results demonstrated that subjectivity had an effect on the duration of the connective, with an estimated mean of 0.05 s (95% CrI [0.03 s, 0.06 s]). The Bayes factors evaluating this effect exceeded 100 for all priors incorporated, indicating very strong support for the presence of the effect. The estimates suggested that the duration of the connective was longer in subjective causality than in objective causality, with an estimated mean difference of 50 ms.

Moreover, the results provided some weak evidence (*BF_10_* ranging from 5.87 to 9.00) for subjectivity’s effect on the FPC2 score of the F0 contour of the connective (Estimate = 0.85, 95% CrI [0.24, 1.46]). This estimation indicated that, on average, FPC2’s weight was 0.85 units higher in instances of subjective causality than in cases of objective causality. In descriptive terms, this means that the shape of the F0 contour of the connective exhibited a more concave pattern in subjective causality compared with objective causality.

In addition, the results presented a blend of evidence concerning the influence of subjectivity on the weight of FPC3, revealing an estimate of −0.09 (95% CrI [−0.19, 0.00]). The model indicated that the weight of FPC3 was reduced by 0.09 units in instances of subjective causality compared with objective causality. Descriptively, this meant that the F0 contour of the connective “so” had a smaller intercept with the y-axis in subjective causality compared with objective causality. However, it is worth noting that when calculated using the most informative prior, that is, normal (0, 0.1), the Bayes factor (*BF_10_*) was 2.00, suggesting that the model containing the effect was twice as favorable as the model not containing the effect. However, when adopting a less informative prior, that is, normal (0, 0.5), the *BF_10_* dropped to 0.62, lending support to the model without the effect of subjectivity.

### 3.6 Interim summary

Our findings revealed several prosodic differences between subjective and objective causality in the forward order. Specifically, we observed that the connective “so” had a longer duration in subjective causality than in objective causality. Subsequent analyses demonstrated that both the consonant and vowel of “so” were lengthened in the subjective condition. The estimated lengthening for the consonant was 15 ms (95% CrI [6 ms, 25 ms], *BF_10_* = 36.2), whereas the vowel exhibited a lengthening of 22 ms (95% CrI [10 ms, 30 ms], *BF_10_* >> 100) in the subjective condition. The implications of these findings will be discussed in the “General discussion” section.

Moreover, we observed that subjective forward causality exhibited a higher F0 maximum, resulting in an expanded F0 range, in comparison with objective forward causality, aligning with our prediction.

In addition to the aforementioned distinctions in the static prosodic measurements, we identified differences between the two types of causality in the F0 trajectory across the connective “so.” However, contrary to our prediction, the distinction was not found in the alignment of the F0 peak. Instead, the disparity was observed in the curvature of the F0 contour of the connective, which exhibited a more concave pattern and a final F0 rise in subjective causality compared with objective causality.

## 4 General discussion

This study aimed to investigate the role of prosody in conveying subjective and objective causality when specialized causal connective words are absent. To achieve this objective, we conducted two experiments examining the production of subjective and objective causality in English, which is commonly expressed using a general causal connective. Experiment 1 specifically examined these two types of causality in a backward causal order, whereas Experiment 2 focused on their expression in a forward causal order.

We hypothesized that prosody would play a role in distinguishing between subjective and objective causality when expressed by a general causal connective that does not differentiate between them. To test the hypothesis, we formulated three specific predictions regarding the prosodic distinctions between subjective and objective causality. First, we predicted that utterances expressing subjective causality may demonstrate less integrated prosody compared with utterances expressing objective causality. The results of Experiment 1 supported this prediction, revealing a longer pause duration before the connective in subjective causality compared with objective causality. This finding suggests that the two clauses involved in subjective causality are more prosodically separated, compared with the two clauses involved in objective causality, indicating a conceptual distinction between the two types of causality as proposed in the discourse literature (Zufferey, 2012). Furthermore, it is known that longer pauses preceding a phrase contribute to the perception of a stronger prosodic boundary before that phrase (Ferreira, 1993; Swerts & Geluykens, 1994). Therefore, our results suggest that complex sentences expressing subjective causality are more likely to be produced as two distinct information units compared with those expressing objective causality, supporting the claims made in discourse literature (Couper-Kuhlen, 1996; Rutherford, 1970; Stukker & Sanders, 2012; Sweetser, 1990). In Experiment 2, although the duration of the pause did not differ between the two types of causality, we observed that the initial consonant in the connective “so” was longer in the subjective condition compared with the objective condition. Previous studies have established that phrasal-initial lengthening can contribute to the perception of a prosodic boundary preceding that phrase (Fougeron & Keating, 1997; Keating et al., 2004). Therefore, our results suggest that subjective forward causality is more likely to exhibit a stronger prosodic boundary preceding the connective compared with objective forward causality, providing potential support for the claim made in previous discourse studies (Couper-Kuhlen, 1996; Rutherford, 1970; Sweetser, 1990). However, it is important to note that our conclusions regarding prosodic boundaries are only preliminary. To validate this conclusion and further investigate the prosodic phrasing in subjective and objective causality, a phonological approach with more materials and the involvement of human annotators is necessary.

Our second prediction posited that subjective causality would be produced with a wider F0 range than objective causality, due to the involvement of an SoC in constructing subjective causality. The results from both experiments supported this prediction.

In addition, we explored the impact of subjectivity on the overall shape of the F0 contour. In the case of forward causality, we observed that the F0 contour of the connective exhibited a more concave shape and a final rise in subjective causality than in objective causality. However, for backward causality, there was only very weak evidence suggesting that the F0 contour of the first clause has a larger final rise in cases of subjective causality than in cases of objective causality. It is important to note that this evidence is not as strong as in the forward causality case. Previous research has linked the concave–convex contour distinction to the perceived degree of harshness or softness in an utterance (Yang & Campbell, 2001). Our findings further extend this finding by demonstrating that this contour distinction is also associated with the subjective–objective contrast in English utterances expressing causality.

Although subjective and objective causality in the forward order show similar prosodic distinctions to those in the backward order, the specific locations where those distinctions were observed differed between forward and backward causality. In backward subjective causality, the expanded F0 range was observed in the first clause, whereas in forward subjective causality, it was observed in the second clause. Given that these clauses convey opinions in subjective causality (whereas the other clauses state facts), our finding suggests that although subjectivity serves to connect the two clauses in complex sentences expressing subjective causality and has an effect on F0, its impact is confined to the clause conveying the opinion rather than spreading over the entire utterance. This finding is consistent with prior research on the prosodic realizations of stance in speech, which showed that words or short sentences conveying stance exhibited a wider F0 range, compared to those expressing neutrality (Freeman, 2014, 2019; Morency et al., 2011). These findings, along with our current results, collectively emphasize that the presence of subjectivity (or stance) in constructing utterances is reflected in their prosodic features. Thus, it is conceivable that, in a given language, utterances expressing subjective causality, particularly the clause expressing opinion, would exhibit prosodic features indicative of how subjective (stance) is expressed through prosody in that language.

The order of causality not only influences the location of the F0 distinction, but also affects the location of the durational difference. We found that backward subjective causality (Experiment 1) featured a longer pause between the main clause and the subordinate clause, whereas forward subjective causality (Experiment 2) exhibited a lengthened connective “so” at the beginning of the subordinate clause. The distinct locations of duration-related differences may be attributed to variations in the syntax–prosody interface between backward and forward constructions. In the backward construction, the “*because*-clause” maintains a subordinating relationship with the main clause and is typically produced without a pause between them (Lelandais & Ferré, 2016; Tyler, 2013). Our results indicate that speakers utilize the acoustic space between the main clause and “because” to encode subjectivity by introducing a longer pause in subjective causality. In the forward construction, the “*so*-clause” and the preceding clause function as coordinated clauses, and are typically produced with a pause between them. Thus, the acoustic space between the first main clause and “so” is employed to mark the syntactic structure regardless of causality. In this case, speakers vary the duration of the first word after the pause, namely the connective “so,” to distinguish between different types of causality. Alternatively, the difference in the location of duration-related difference can be attributed to the order of *P* and *Q*. In backward subjective causality, *Q* precedes *P*, which contrasts with the natural order of events. Consequently, this creates a discontinuity in mental representation and thus a temporal gap in speech production. Conversely, in forward subjective causality, *P* precedes *Q*, aligning with the natural order of events, resulting in no acoustic gap between *P* and *Q* during production.

In summary, our study identified several prosodic differences between subjective and objective causality in English in the absence of specific lexical markers. Subjective causality exhibited an expanded F0 range, less integrated prosody, and distinct F0 contour shapes, compared with objective causality, with the specific locations of these features varying depending on the order of causality.

The prosodic distinction between subjective and objective causality corresponds to their cognitive distinctions. The expanded F0 range associated with subjective causality reflects the involvement of an SoC in its construction. Moreover, the extended duration observed in subjective causality aligns with the notion that subjective causality is cognitively more complex compared with objective causality (Traxler et al., 1997; Zufferey & Gygax, 2015). In addition, the less integrated prosody in subjective causality supports the idea that the meanings of *P* and *Q* are more distantly related in subjective causality compared with objective causality (Sweetser, 1990; Verhagen, 2005; Zufferey, 2012).

Our results contribute further to the research on the prosodic realizations of lexico-grammatical expressions and their associated functions and meanings. Prior studies have shown that words or phrases with multiple functions display different prosodic profiles corresponding to those functions. For instance, Wichmann (2005) has shown that the word “please” displays different intonational patterns depending on its functions. In addition, Dehé and Wichmann (2010) have shown that expressions such as “I think (that)” and “I believe (that)” exhibit different prosodic patterns between grammatical functions as the main clause, comment clause, or discourse marker. Our current findings reveal that the causal connective “so” shows different prosodic features for subjective and objective causality in the forward order, with the former exhibiting longer duration and a concave (rising) F0 contour. This suggests that the prosodic profile of this discourse marker varies depending on the type of causality it is used to express. This finding, along with previous studies, collectively indicate that the prosodic realization of a word or phrase is associated with its function or meaning.

One important question arising from our findings concerns the perceptual relevance of the observed prosodic differences between subjective and objective causality—namely, whether listeners can distinguish between the two types of causality based solely on prosodic cues, in the absence of morpho-syntactic markers. This question was addressed in the study by Hu et al. (2023), who examined the effect of the prosody of the connective “so” using a forced-choice discourse completion task. In the task, the participants first listened to audio stimuli describing real-world events that ended with the connective “so” (e.g., “*Jim got his nose pierced so*”), where “so” was pronounced with either subjective causality prosody (i.e., longer duration and a concave f0 contour) or objective causality prosody, constructed based on model estimates from this study. After listening to each stimulus, participants were asked to choose between two continuations displayed on the screen: one establishing subjective causality with the stimulus and the other objective causality. The results showed that the prosody of “so” had an effect on participants’ choices. When “so” was produced with prosody typical of subjective causality, participants (on average) were 1.48 times more likely to select the continuation reflecting a subjective interpretation, compared to when “so” was produced with objective causality prosody.

Furthermore, regarding the causal connective “so,” although perceptual evidence is still needed, it is conceivable that it would be perceived as prominent in subjective causality due to its prolonged duration, which is a strong indicator of prosodic prominence. Thus, this could suggest that prosodic prominence is associated with subjectivity, aligning with previous research. For example, Aijmer (2011) argues that when receiving prosodic prominence, “I think” functions as a discourse marker conveying certainty, but is more tentative when lacking prominence. Similarly, Kärkkäinen (2003) proposes that “I think” serves subjective (or epistemic) functions in natural conversation when it is prosodically prominent but functions as a discourse boundary marker when not prominent. Furthermore, Wichmann et al. (2010) argue that a single adverbial—“of course”—is more likely to be marked with prosodic prominence when it serves epistemic stance functions. These findings collectively suggest that, in addition to marking information prominence (Bolinger, 1978; Cutler et al., 1997), prosodic prominence can convey subjectivity (stance) in conversation.

This study paves the way for further research on this topic by posing several important follow-up questions. For instance, it is important to explore whether other acoustic cues, such as voice quality and amplitude, also contribute to distinguishing between the two types of causality, as these cues are often involved in marking linguistic contrasts (e.g., stance, Freese & Maynard, 1998). In addition, future research should investigate whether prosody continues to play a role in expressing subjective and objective causality when specialized causal connectives are used. It is also crucial to examine whether the patterns observed in English extend to other languages, or if they vary due to differences in prosodic systems. This is an important empirical issue that can only be addressed through cross-linguistic research. Although languages may share general causal connectives, their prosody could be similar to or different from English, potentially leading to different patterns in the expression of causality. This study introduces an experimental paradigm that can be applied to cross-linguistic research, allowing us to explore these questions and gain a deeper understanding of the interplay between prosody, causality, and comprehension across languages.

## 5 Conclusion

This study aimed to investigate the prosodic distinctions between subjective and objective causality in English, two cognitively distinct yet lexically underspecified causal relations. Two production experiments were conducted, focusing on backward and forward causality, respectively. The findings unveiled prosodic differences between subjective and objective causality. Specifically, subjective causality was characterized by a larger F0 range, less integrated prosody, and distinct F0 contour shapes, compared with objective causality. These results emphasize that prosodic means are employed to express the distinction between subjective and objective causality in the absence of explicit lexical markers.

## Supplemental Material

sj-docx-1-las-10.1177_00238309251369482 – Supplemental material for The Role of Prosody in Expressing Subjective and Objective Causality in English Supplemental material, sj-docx-1-las-10.1177_00238309251369482 for The Role of Prosody in Expressing Subjective and Objective Causality in English by Na Hu, Aoju Chen, Hugo Quené and Ted J. M. Sanders in Language and Speech

sj-docx-2-las-10.1177_00238309251369482 – Supplemental material for The Role of Prosody in Expressing Subjective and Objective Causality in English Supplemental material, sj-docx-2-las-10.1177_00238309251369482 for The Role of Prosody in Expressing Subjective and Objective Causality in English by Na Hu, Aoju Chen, Hugo Quené and Ted J. M. Sanders in Language and Speech

sj-docx-3-las-10.1177_00238309251369482 – Supplemental material for The Role of Prosody in Expressing Subjective and Objective Causality in English Supplemental material, sj-docx-3-las-10.1177_00238309251369482 for The Role of Prosody in Expressing Subjective and Objective Causality in English by Na Hu, Aoju Chen, Hugo Quené and Ted J. M. Sanders in Language and Speech
