# Supplementary material for: The Role of Prosody in Expressing Subjective and Objective Causality in English
Source: Lang Speech. 2025 Nov 2;69(3):753–77. doi: 10.1177/00238309251369482 (PMC13494113; doi:10.1177/00238309251369482)
Supplement: Supplementary file 1 [file sj-docx-1-las-10.1177_00238309251369482.docx]

# Instructions

The participants were provided with the subsequent instructions before engaging in the task for Experiment 1. The instructions given for Experiment 2 remain unchanged from those of Experiment 1, except that the two optional connectives are “so” and “but”.

You are going to read some slides. After each slide, you will get three questions from your conversation partner. Please formulate your answers in the following way.

Use the first sentence to answer the first question. Use the second and third sentences to answer the second question. Combine these two sentences with either “because” or “however”. Use the last sentence to answer the last question. Please read information on the slides carefully. Do not change or add words. Do not alter the order of sentences. Please produce your answers naturally.
