# Supplementary material for: The Role of Prosody in Expressing Subjective and Objective Causality in English
Source: Lang Speech. 2025 Nov 2;69(3):753–77. doi: 10.1177/00238309251369482 (PMC13494113; doi:10.1177/00238309251369482)
Supplement: Supplementary file 2 [file sj-docx-2-las-10.1177_00238309251369482.docx]

# Statistical summaries

These summaries serve as supplements to Sections 2.7 and 3.5, presenting full model summaries. Each table within this section encompasses the prior probabilities employed for computing Bayes factors that assess the existence or absence of each effect. Additionally, the tables include the derived Bayes factors, along with the mean and the 95% credibility interval of the posterior distribution for each effect. The titles of the tables correspond to the respective dependent variables.

**A. Experiment 1**

Table 1

*Pause duration*

| Model terms | Prior | *BF_10_* | Posterior | | |
| --- | --- | --- | --- | --- | --- |
|  |  |  | Estimate | Lower | Upper |
| (1+Subjectivity\|item) | normal(0, 5) | 0.02 | 0.04 | 0.00 | 0.08 |
|  | normal(0, 2) | 0.04 | 0.04 | 0.00 | 0.08 |
|  | normal(0, 1) | 0.08 | 0.04 | 0.00 | 0.08 |
| (1+Subjectivity\|subject) | normal(0, 10) | 0.73 | 0.05 | 0.02 | 0.08 |
|  | normal(0, 8) | 0.92 | 0.05 | 0.02 | 0.08 |
|  | normal(0, 6) | 1.28 | 0.05 | 0.02 | 0.08 |
| gender: subjectivity | normal(0, 0.1) | 0.28 | 0.01 | -0.04 | 0.06 |
|  | normal(0, 0.3) | 0.10 | 0.01 | -0.04 | 0.06 |
|  | normal(0, 0.5) | 0.06 | 0.01 | -0.04 | 0.06 |
| subjectivity | normal(0, 0.05) | 40.88 | 0.05 | 0.02 | 0.07 |
|  | normal(0, 0.1) | 29.48 | 0.05 | 0.02 | 0.08 |
|  | normal(0, 0.2) | 16.48 | 0.05 | 0.02 | 0.08 |
| gender | normal(0, 0.1) | 0.49 | 0.01 | -0.09 | 0.10 |
|  | normal(0, 0.3) | 0.17 | 0.01 | -0.10 | 0.12 |
|  | normal(0, 0.5) | 0.11 | 0.01 | -0.10 | 0.13 |

Table 2

*Connective duration*

| Model terms | Prior | *BF_10_* | Posterior | | |
| --- | --- | --- | --- | --- | --- |
|  |  |  | Estimate | Lower | Upper |
| (1+Subjectivity\|item) | normal(0, 5) | 0.01 | 0.02 | 0.00 | 0.04 |
|  | normal(0, 2) | 0.02 | 0.02 | 0.00 | 0.04 |
|  | normal(0, 1) | 0.03 | 0.02 | 0.00 | 0.04 |
| (1+Subjectivity\|subject) | normal(0, 5) | 0.002 | 0.01 | 0.00 | 0.02 |
|  | normal(0, 2) | 0.006 | 0.01 | 0.00 | 0.02 |
|  | normal(0, 1) | 0.01 | 0.01 | 0.00 | 0.02 |
| gender: subjectivity | normal(0, 0.1) | 0.14 | -0.00 | -0.03 | 0.02 |
|  | normal(0, 0.3) | 0.05 | -0.00 | -0.03 | 0.02 |
|  | normal(0, 0.5) | 0.03 | -0.00 | -0.03 | 0.02 |
| subjectivity | normal(0, 0.1) | 0.08 | -0.00 | -0.02 | 0.01 |
|  | normal(0, 0.3) | 0.03 | -0.00 | -0.02 | 0.01 |
|  | Normal(0, 0.5) | 0.02 | -0.00 | -0.02 | 0.01 |
| gender | Normal(0, 0.1) | 0.73 | 0.04 | -0.03 | 0.11 |
|  | Normal(0, 0.3) | 0.28 | 0.05 | -0.03 | 0.12 |
|  | Normal(0, 0.5) | 0.16 | 0.05 | -0.03 | 0.12 |

Table 3

*F0 contours of the connective ‘because’*

a. FPC1

| Model terms | Prior | *BF_10_* | Posterior | | |
| --- | --- | --- | --- | --- | --- |
|  |  |  | Estimate | Lower | Upper |
| (1+Subjectivity\|item) | normal(0, 5) | 0.57 | 1.95 | 0.09 | 4.57 |
|  | normal(0, 2) | 0.91 | 1.43 | 0.06 | 3.69 |
|  | normal(0, 1) | 0.98 | 0.78 | 0.03 | 2.21 |
| (1+Subjectivity\|subject) | normal(0, 10) | 2.46 | 2.51 | 0.62 | 4.77 |
|  | normal(0, 8) | 2.92 | 2.48 | 0.59 | 4.74 |
|  | normal(0, 6) | 3.90 | 2.46 | 0.56 | 4.67 |
| gender: subjectivity | normal(0, 5) | 0.58 | -1.73 | -5.23 | 1.75 |
|  | normal(0, 10) | 0.31 | -1.91 | -5.60 | 1.80 |
|  | normal(0, 15) | 0.22 | -1.92 | -5.63 | 1.86 |
| subjectivity | normal(0, 1) | 0.67 | -0.16 | -1.46 | 1.13 |
|  | normal(0, 1.5) | 0.53 | -0.21 | -1.69 | 1.30 |
|  | normal(0, 2) | 0.41 | -0.24 | -1.83 | 1.35 |
| gender | normal(0, 5) | 1.02 | 2.84 | -2.27 | 7.60 |
|  | normal(0, 10) | 0.67 | 3.49 | -2.21 | 9.12 |
|  | normal(0, 15) | 0.47 | 3.68 | -2.23 | 9.46 |

b. FPC2

| Model terms | Prior | *BF_10_* | Posterior | | |
| --- | --- | --- | --- | --- | --- |
|  |  |  | Estimate | Lower | Upper |
| (1+Subjectivity\|item) | normal(0, 5) | 0.43 | 1.26 | 0.07 | 2.86 |
|  | normal(0, 2) | 0.88 | 1.10 | 0.05 | 2.58 |
|  | normal(0, 1) | 1.14 | 0.78 | 0.03 | 1.98 |
| (1+Subjectivity\|subject) | normal(0, 5) | 0.14 | 0.56 | 0.02 | 1.63 |
|  | normal(0, 2) | 0.34 | 0.54 | 0.02 | 1.52 |
|  | normal(0, 1) | 0.59 | 0.46 | 0.02 | 1.31 |
| gender: subjectivity | normal(0, 5) | 0.53 | 1.53 | -0.82 | 3.93 |
|  | normal(0, 10) | 0.29 | 1.60 | -0.80 | 4.02 |
|  | normal(0, 15) | 0.19 | 1.61 | -0.82 | 4.02 |
| subjectivity | normal(0, 1) | 1.10 | -0.58 | -1.50 | 0.36 |
|  | normal(0, 1.5) | 0.84 | -0.67 | -1.67 | 0.34 |
|  | normal(0, 2) | 0.68 | -0.70 | -1.74 | 0.34 |
| gender | normal(0, 5) | 0.39 | -0.32 | -4.40 | 3.80 |
|  | normal(0, 10) | 0.21 | -0.32 | -4.81 | 4.12 |
|  | normal(0, 15) | 0.15 | -0.35 | -4.86 | 4.22 |

c. FPC3

| Model terms | Prior | *BF_10_* | Posterior | | |
| --- | --- | --- | --- | --- | --- |
|  |  |  | Estimate | Lower | Upper |
| (1+Subjectivity\|item) | normal(0, 5) | 0.09 | 0.77 | 0.03 | 1.97 |
|  | normal(0, 2) | 0.19 | 0.72 | 0.03 | 1.83 |
|  | normal(0, 1) | 0.31 | 0.59 | 0.02 | 1.56 |
| (1+Subjectivity\|subject) | normal(0, 5) | 0.05 | 0.47 | 0.02 | 1.39 |
|  | normal(0, 2) | 0.11 | 0.45 | 0.02 | 1.31 |
|  | normal(0, 1) | 0.20 | 0.41 | 0.02 | 1.17 |
| gender: subjectivity | Normal(0, 5) | 0.08 | 0.24 | -1.71 | 2.19 |
|  | Normal(0, 10) | 0.04 | 0.25 | -1.74 | 2.25 |
|  | Normal(0, 15) | 0.03 | 0.23 | -1.79 | 2.25 |
| subjectivity | Normal(0, 0.1) | 1.01 | -0.03 | -0.22 | 0.16 |
|  | Normal(0, 0.3) | 1.04 | -0.17 | -0.64 | 0.30 |
|  | Normal(0, 0.5) | 0.97 | -0.28 | -0.89 | 0.33 |
| gender | Normal(0, 5) | 0.21 | -0.30 | -2.44 | 1.86 |
|  | Normal(0, 10) | 0.11 | -0.33 | -2.56 | 1.88 |
|  | Normal(0, 15) | 0.07 | -0.32 | -2.55 | 1.89 |

Table 4

*F0*

a. C1f0Maxst

| Model terms | Prior | *BF_10_* | Posterior | | |
| --- | --- | --- | --- | --- | --- |
|  |  |  | Estimate | Lower | Upper |
| (1+Subjectivity\|item) | normal(0, 10) | 0.11 | 0.62 | 0.03 | 1.31 |
|  | normal(0, 5) | 0.20 | 0.62 | 0.03 | 1.31 |
|  | normal(0, 2) | 0.49 | 0.61 | 0.03 | 1.28 |
| (1+Subjectivity\|subject) | normal(0, 10) | 0.15 | 0.53 | 0.04 | 1.13 |
|  | normal(0, 5) | 0.29 | 0.53 | 0.05 | 1.13 |
|  | normal(0, 2) | 0.71 | 0.52 | 0.04 | 1.09 |
| gender: subjectivity | Normal (0, 1) | 0.48 | 0.21 | -0.57 | 0.99 |
|  | Normal (0, 1.5) | 0.33 | 0.23 | -0.57 | 1.04 |
|  | Normal (0, 2) | 0.23 | 0.24 | -0.59 | 1.07 |
| subjectivity | Normal(0, 0.5) | 7.19 | 0.50 | 0.09 | 0.90 |
|  | Normal(0, 1) | 5.87 | 0.58 | 0.14 | 1.01 |
|  | Normal(0, 1.5) | 4.70 | 0.59 | 0.14 | 1.04 |
| gender | Normal(0, 15) | 36.06 | -11.12 | -17.17 | -4.80 |
|  | Normal(0, 20) | 31.47 | -11.39 | -17.49 | -5.14 |
|  | Normal(0, 25) | 27.32 | -11.50 | -17.75 | -5.22 |

b. C1f0Minst

| Model terms | Prior | *BF_10_* | Posterior | | |
| --- | --- | --- | --- | --- | --- |
|  |  |  | Estimate | Lower | Upper |
| (1+Subjectivity\|item) | normal(0, 10) | 0.10 | 0.63 | 0.03 | 1.36 |
|  | normal(0, 5) | 0.21 | 0.64 | 0.03 | 1.37 |
|  | normal(0, 2) | 0.45 | 0.62 | 0.03 | 1.35 |
| (1+Subjectivity\|subject) | normal(0, 10) | 0.04 | 0.28 | 0.01 | 0.74 |
|  | normal(0, 5) | 0.08 | 0.28 | 0.01 | 0.74 |
|  | normal(0, 2) | 0.18 | 0.28 | 0.01 | 0.73 |
| gender: subjectivity | Normal (0, 1) | 0.71 | 0.43 | -0.38 | 1.25 |
|  | Normal (0, 1.5) | 0.53 | 0.48 | -0.37 | 1.33 |
|  | Normal (0, 2) | 0.42 | 0.50 | -0.37 | 1.38 |
| subjectivity | Normal(0, 0.5) | 13.04 | -0.58 | -1.00 | -0.15 |
|  | Normal(0, 1) | 12.75 | -0.67 | -1.12 | -0.21 |
|  | Normal(0, 1.5) | 10.20 | -0.69 | -1.15 | -0.23 |
| gender | Normal(0, 15) | >>100 | -11.50 | -15.05 | -7.89 |
|  | Normal(0, 20) | >>100 | -11.53 | -15.09 | -7.89 |
|  | Normal(0, 25) | >>100 | -11.61 | -15.17 | -7.98 |

c. C2f0Maxst

| Model terms | Prior | *BF_10_* | Posterior | | |
| --- | --- | --- | --- | --- | --- |
|  |  |  | Estimate | Lower | Upper |
| (1+Subjectivity\|item) | normal(0, 5) | 0.24 | 0.90 | 0.03 | 1.89 |
|  | normal(0, 2) | 0.54 | 0.86 | 0.03 | 1.83 |
|  | normal(0, 1) | 0.78 | 0.69 | 0.02 | 1.65 |
| (1+Subjectivity\|subject) | normal(0, 5) | 0.04 | 0.20 | 0.01 | 0.60 |
|  | normal(0, 2) | 0.12 | 0.20 | 0.01 | 0.59 |
|  | normal(0, 1) | 0.23 | 0.20 | 0.01 | 0.57 |
| gender: subjectivity | normal (0, 1) | 0.48 | 0.21 | -0.61 | 1.04 |
|  | normal (0, 1.5) | 0.35 | 0.24 | -0.63 | 1.09 |
|  | normal (0, 2) | 0.25 | 0.25 | -0.65 | 1.14 |
| subjectivity | normal(0, 1) | 0.35 | 0.17 | -0.40 | 0.74 |
|  | normal(0, 1.5) | 0.23 | 0.18 | -0.42 | 0.77 |
|  | normal(0, 2) | 0.17 | 0.18 | -0.41 | 0.78 |
| gender | normal(0, 15) | >>100 | -11.82 | -16.71 | -6.68 |
|  | normal(0, 20) | >>100 | -11.93 | -16.79 | -6.96 |
|  | normal(0, 25) | >>100 | -11.94 | -16.89 | -6.95 |

d. C2f0Minst

| Model terms | Prior | *BF_10_* | Posterior | | |
| --- | --- | --- | --- | --- | --- |
|  |  |  | Estimate | Lower | Upper |
| (1+Subjectivity\|item) | normal(0, 5) | 0.41 | 1.02 | 0.07 | 2.06 |
|  | normal(0, 2) | 0.96 | 0.94 | 0.05 | 1.97 |
|  | normal(0, 1) | 1.38 | 0.77 | 0.04 | 1.74 |
| (1+Subjectivity\|subject) | normal(0, 10) | 0.33 | 0.88 | 0.11 | 1.77 |
|  | normal(0, 8) | 0.82 | 0.88 | 0.10 | 1.75 |
|  | normal(0, 6) | 1.04 | 0.86 | 0.10 | 1.73 |
| gender: subjectivity | Normal (0, 1) | 0.78 | 0.47 | -0.60 | 1.53 |
|  | Normal (0, 1.5) | 0.63 | 0.56 | -0.61 | 1.69 |
|  | Normal (0, 2) | 0.49 | 0.60 | -0.60 | 1.78 |
| subjectivity | Normal(0, 1) | 0.84 | -0.44 | -1.10 | 0.23 |
|  | Normal(0, 1.5) | 0.63 | -0.47 | -1.15 | 0.22 |
|  | Normal(0, 2) | 0.48 | -0.48 | -1.17 | 0.21 |
| gender | Normal(0, 15) | >>100 | -11.51 | -15.01 | -7.95 |
|  | Normal(0, 20) | >>100 | -11.59 | -15.07 | -8.02 |
|  | Normal(0, 25) | >>100 | -11.60 | -15.07 | -8.16 |

Table 5

Articulation rate

a. C1AR

| Model terms | Prior | *BF_10_* | Posterior | | |
| --- | --- | --- | --- | --- | --- |
|  |  |  | Estimate | Lower | Upper |
| (1+Subjectivity\|item) | normal(0, 5) | 0.01 | 1.00 | 0.04 | 1.94 |
|  | normal(0, 2) | 0.003 | 0.92 | 0.04 | 1.89 |
|  | normal(0, 1) | 0.07 | 0.74 | 0.03 | 1.74 |
| (1+Subjectivity\|subject) | normal(0, 5) | 0.10 | 0.17 | 0.02 | 0.35 |
|  | normal(0, 2) | 0.26 | 0.17 | 0.02 | 0.36 |
|  | normal(0, 1) | 0.50 | 0.17 | 0.02 | 0.36 |
| gender: subjectivity | Normal (0, 0.1) | 0.82 | -0.00 | -0.16 | 0.16 |
|  | Normal (0, 0.3) | 0.43 | -0.01 | -0.26 | 0.25 |
|  | Normal (0, 0.5) | 0.27 | -0.01 | -0.28 | 0.26 |
| subjectivity | Normal(0, 1) | 0.37 | 0.20 | -0.34 | 0.75 |
|  | Normal(0, 1.5) | 0.26 | 0.21 | -0.36 | 0.78 |
|  | Normal(0, 2) | 0.19 | 0.21 | -0.37 | 0.79 |
| gender | Normal(0, 1) | 0.40 | 0.13 | -0.68 | 0.91 |
|  | Normal(0, 1.5) | 0.29 | 0.14 | -0.71 | 0.99 |
|  | Normal(0, 2) | 0.23 | 0.14 | -0.75 | 1.02 |

b. C2 AR

| Model terms | Prior | *BF_10_* | Posterior | | |
| --- | --- | --- | --- | --- | --- |
|  |  |  | Estimate | Lower | Upper |
| (1+Subjectivity\|item) | normal(0, 5) | 0.009 | 0.93 | 0.04 | 1.81 |
|  | normal(0, 2) | 0.0004 | 0.89 | 0.03 | 1.79 |
|  | normal(0, 1) | 1.83 | 0.67 | 0.02 | 1.63 |
| (1+Subjectivity\|subject) | normal(0, 5) | 0.02 | 0.09 | 0.00 | 0.25 |
|  | normal(0, 2) | 0.06 | 0.09 | 0.00 | 0.25 |
|  | normal(0, 1) | 0.13 | 0.09 | 0.00 | 0.25 |
| gender: subjectivity | Normal (0, 0.1) | 0.88 | -0.03 | -0.19 | 0.13 |
|  | Normal (0, 0.3) | 0.50 | -0.08 | -0.32 | 0.17 |
|  | Normal (0, 0.5) | 0.32 | -0.08 | -0.35 | 0.18 |
| subjectivity | Normal(0, 1) | 0.27 | -0.02 | -0.55 | 0.50 |
|  | Normal(0, 1.5) | 0.18 | -0.03 | -0.56 | 0.50 |
|  | Normal(0, 2) | 0.13 | -0.03 | -0.55 | 0.51 |
| gender | Normal(0, 1) | 0.45 | -0.07 | -1.02 | 0.89 |
|  | Normal(0, 1.5) | 0.34 | -0.07 | -1.10 | 0.97 |
|  | Normal(0, 2) | 0.26 | -0.07 | -1.13 | 1.01 |

Table 6

*F0 contours of the first clause*

a. FPC1

| Model terms | Prior | *BF_10_* | Posterior | | |
| --- | --- | --- | --- | --- | --- |
|  |  |  | Estimate | Lower | Upper |
| (1+Subjectivity\|item) | normal(0, 20) | 2.31 | 6.40 | 0.36 | 14.45 |
|  | normal(0, 8) | 1.15 | 1.86 | 0.08 | 5.08 |
|  | normal(0, 6) | 1.04 | 0.83 | 0.03 | 2.32 |
| (1+Subjectivity\|subject) | normal(0, 5) | 0.68 | 2.73 | 0.10 | 7.74 |
|  | normal(0, 2) | 0.90 | 1.45 | 0.05 | 4.13 |
|  | normal(0, 1) | 0.98 | 0.78 | 0.03 | 2.20 |
| gender: subjectivity | Normal (0, 5) | 0.84 | 1.06 | -7.12 | 9.18 |
|  | Normal (0, 10) | 0.63 | 2.26 | -9.45 | 14.00 |
|  | Normal (0, 15) | 0.47 | 2.78 | -10.40 | 15.67 |
| subjectivity | Normal(0, 1) | 1.06 | -0.28 | -2.22 | 1.66 |
|  | Normal(0, 1.5) | 1.07 | -0.62 | -3.46 | 2.17 |
|  | Normal(0, 2) | 1.12 | -1.07 | -4.76 | 2.62 |
| gender | Normal(0, 5) | 0.97 | 0.01 | -9.50 | 9.66 |
|  | Normal(0, 10) | 0.89 | 0.11 | -17.30 | 17.59 |
|  | Normal(0, 15) | 0.79 | 0.15 | -22.98 | 23.65 |

b. FPC2

| Model terms | Prior | *BF_10_* | Posterior | | |
| --- | --- | --- | --- | --- | --- |
|  |  |  | Estimate | Lower | Upper |
| (1+Subjectivity\|item) | normal(0, 5) | 0.90 | 3.65 | 0.14 | 10.66 |
|  | normal(0, 2) | 0.98 | 1.56 | 0.06 | 4.45 |
|  | normal(0, 1) | 0.99 | 0.79 | 0.03 | 2.22 |
| (1+Subjectivity\|subject) | normal(0, 5) | 1.19 | 3.98 | 0.19 | 9.76 |
|  | normal(0, 2) | 1.12 | 1.72 | 0.07 | 4.67 |
|  | normal(0, 1) | 1.02 | 0.81 | 0.03 | 2.31 |
| gender: subjectivity | Normal (0, 20) | 0.43 | 4.05 | -10.17 | 18.24 |
|  | Normal (0, 30) | 0.30 | 4.44 | -10.76 | 19.58 |
|  | Normal (0, 50) | 0.18 | 4.63 | -10.58 | 19.85 |
| subjectivity | Normal(0, 5) | 1.08 | -3.29 | -10.54 | 4.24 |
|  | Normal(0, 10) | 0.94 | -5.56 | -15.11 | 4.08 |
|  | Normal(0, 15) | 0.75 | -6.38 | -16.66 | 3.96 |
| gender | Normal(0, 20) | 0.52 | 5.81 | -8.54 | 20.14 |
|  | Normal(0, 30) | 0.36 | 6.23 | -8.70 | 21.21 |
|  | Normal(0, 50) | 0.23 | 6.47 | -9.05 | 21.88 |

c. FPC3

| Model terms | Prior | *BF_10_* | Posterior | | |
| --- | --- | --- | --- | --- | --- |
|  |  |  | Estimate | Lower | Upper |
| (1+Subjectivity\|item) | normal(0, 5) | 1.29 | 4.66 | 0.19 | 11.80 |
|  | normal(0, 2) | 1.07 | 1.67 | 0.06 | 4.71 |
|  | normal(0, 1) | 1.04 | 0.82 | 0.04 | 2.29 |
| (1+Subjectivity\|subject) | normal(0, 5) | 1.03 | 0.82 | 0.03 | 2.25 |
|  | normal(0, 2) | 1.02 | 0.82 | 0.03 | 2.28 |
|  | normal(0, 1) | 1.02 | 0.81 | 0.03 | 2.28 |
| gender: subjectivity | Normal (0, 20) | 0.35 | -0.53 | -14.29 | 13.29 |
|  | Normal (0, 30) | 0.24 | -0.60 | -14.70 | 13.61 |
|  | Normal (0, 50) | 0.15 | -0.59 | -14.89 | 13.94 |
| subjectivity | Normal(0, 5) | 0.58 | -0.50 | -6.16 | 5.22 |
|  | Normal(0, 10) | 0.34 | -0.70 | -7.36 | 5.92 |
|  | Normal(0, 15) | 0.24 | -0.69 | -7.74 | 6.31 |
| gender | Normal(0, 10) | 0.74 | 2.88 | -10.77 | 16.42 |
|  | Normal(0, 15) | 0.61 | 4.01 | -12.17 | 20.11 |
|  | Normal(0, 20) | 0.50 | 4.52 | -13.42 | 22.03 |

**B. Experiment 2**

Table 7

*Pause duration*

| Model terms | Prior | *BF_10_* | Posterior | | |
| --- | --- | --- | --- | --- | --- |
|  |  |  | Estimate | Lower | Upper |
| (1+Subjectivity\|item) | normal(0, 5) | 0.003 | 0.01 | 0.00 | 0.03 |
|  | normal(0, 2) | 0.008 | 0.01 | 0.00 | 0.03 |
|  | normal(0, 1) | 0.02 | 0.01 | 0.00 | 0.03 |
| (1+Subjectivity\|subject) | normal(0, 5) | 0.003 | 0.01 | 0.00 | 0.03 |
|  | normal(0, 2) | 0.007 | 0.01 | 0.00 | 0.03 |
|  | normal(0, 1) | 0.014 | 0.01 | 0.00 | 0.03 |
| gender: subjectivity | Normal(0, 0.1) | 0.17 | 0.01 | -0.03 | 0.04 |
|  | Normal(0, 0.3) | 0.06 | 0.01 | -0.03 | 0.04 |
|  | Normal(0, 0.5) | 0.03 | 0.01 | -0.03 | 0.04 |
| subjectivity | Normal(0, 0.1) | 0.14 | -0.01 | -0.02 | 0.01 |
|  | Normal(0, 0.3) | 0.05 | -0.01 | -0.02 | 0.01 |
|  | Normal(0, 0.5) | 0.03 | -0.01 | -0.02 | 0.01 |
| gender | Normal(0, 0.1) | 0.42 | 0.02 | -0.03 | 0.07 |
|  | Normal(0, 0.3) | 0.15 | 0.03 | -0.03 | 0.08 |
|  | Normal(0, 0.5) | 0.09 | 0.03 | -0.03 | 0.08 |

Table 8

*Connective duration*

| Model terms | Prior | *BF_10_* | Posterior | | |
| --- | --- | --- | --- | --- | --- |
|  |  |  | Estimate | Lower | Upper |
| (1+Subjectivity\|item) | normal(0, 5) | 0.005 | 0.02 | 0.00 | 0.04 |
|  | normal(0, 2) | 0.01 | 0.02 | 0.00 | 0.04 |
|  | normal(0, 1) | 0.03 | 0.02 | 0.00 | 0.04 |
| (1+Subjectivity\|subject) | normal(0, 5) | 0.09 | 0.03 | 0.01 | 0.05 |
|  | normal(0, 2) | 0.22 | 0.03 | 0.01 | 0.05 |
|  | normal(0, 1) | 0.43 | 0.03 | 0.01 | 0.05 |
| gender: subjectivity | Normal(0, 0.1) | 0.16 | 0.00 | -0.03 | 0.03 |
|  | Normal(0, 0.3) | 0.05 | 0.00 | -0.03 | 0.04 |
|  | Normal(0, 0.5) | 0.03 | 0.00 | -0.03 | 0.03 |
| subjectivity | Normal(0, 0.05) | >>100 | 0.05 | 0.03 | 0.06 |
|  | Normal(0, 0.1) | >>100 | 0.05 | 0.03 | 0.06 |
|  | Normal(0, 0.15) | >>100 | 0.05 | 0.03 | 0.07 |
| gender | Normal(0, 0.1) | 0.59 | 0.03 | -0.06 | 0.12 |
|  | Normal(0, 0.3) | 0.23 | 0.04 | -0.06 | 0.14 |
|  | Normal(0, 0.5) | 0.14 | 0.04 | -0.07 | 0.15 |

Table 9

*F0 contours of the connective so*

a. FPC1

| Model terms | Prior | *BF_10_* | Posterior | | |
| --- | --- | --- | --- | --- | --- |
|  |  |  | Estimate | Lower | Upper |
| (1+Subjectivity\|item) | normal(0, 5) | 0.30 | 1.00 | 0.05 | 2.26 |
|  | normal(0, 2) | 0.66 | 0.91 | 0.04 | 2.12 |
|  | normal(0, 1) | 0.92 | 0.70 | 0.03 | 1.78 |
| (1+Subjectivity\|subject) | normal(0, 5) | 0.16 | 0.59 | 0.03 | 1.59 |
|  | normal(0, 2) | 0.39 | 0.57 | 0.02 | 1.51 |
|  | normal(0, 1) | 0.68 | 0.50 | 0.03 | 1.32 |
| gender: subjectivity | Normal(0, 5) | 0.13 | -0.07 | -1.33 | 1.20 |
|  | Normal(0, 10) | 0.06 | -0.07 | -1.33 | 1.20 |
|  | Normal(0, 15) | 0.04 | -0.07 | -1.33 | 1.20 |
| subjectivity | Normal(0, 1) | 0.39 | -0.09 | -0.83 | 0.65 |
|  | Normal(0, 1.5) | 0.27 | -0.10 | -0.87 | 0.68 |
|  | Normal(0, 2) | 0.20 | -0.09 | -0.88 | 0.70 |
| gender | Normal(0, 5) | 2.40 | 1.54 | 0.28 | 2.75 |
|  | Normal(0, 10) | 1.26 | 1.57 | 0.30 | 2.83 |
|  | Normal(0, 15) | 0.84 | 1.57 | 0.31 | 2.82 |

b. FPC2

| Model terms | Prior | *BF_10_* | Posterior | | |
| --- | --- | --- | --- | --- | --- |
|  |  |  | Estimate | Lower | Upper |
| (1+Subjectivity\|item) | normal(0, 5) | 0.15 | 0.55 | 0.02 | 1.46 |
|  | normal(0, 2) | 0.36 | 0.53 | 0.02 | 1.39 |
|  | normal(0, 1) | 0.60 | 0.47 | 0.02 | 1.25 |
| (1+Subjectivity\|subject) | normal(0, 5) | 0.26 | 0.64 | 0.04 | 1.53 |
|  | normal(0, 2) | 0.61 | 0.63 | 0.04 | 1.48 |
|  | normal(0, 1) | 1.08 | 0.56 | 0.03 | 1.32 |
| gender: subjectivity | Normal(0, 5) | 0.13 | 0.26 | -0.93 | 1.42 |
|  | Normal(0, 10) | 0.07 | 0.26 | -0.90 | 1.44 |
|  | Normal(0, 15) | 0.05 | 0.27 | -0.92 | 1.46 |
| subjectivity | Normal(0, 1) | 9.00 | 0.79 | 0.19 | 1.38 |
|  | Normal(0, 1.5) | 7.19 | 0.83 | 0.23 | 1.43 |
|  | Normal(0, 2) | 5.87 | 0.85 | 0.24 | 1.46 |
| gender | Normal(0, 5) | 0.19 | -0.53 | -2.09 | 1.05 |
|  | Normal(0, 10) | 0.09 | -0.53 | -2.10 | 1.07 |
|  | Normal(0, 15) | 0.07 | -0.55 | -2.17 | 1.05 |

c. FPC3

| Model terms | Prior | *BF_10_* | Posterior | | |
| --- | --- | --- | --- | --- | --- |
|  |  |  | Estimate | Lower | Upper |
| (1+Subjectivity\|item) | normal(0, 5) | 0.008 | 0.10 | 0.00 | 0.26 |
|  | normal(0, 2) | 0.02 | 0.10 | 0.00 | 0.26 |
|  | normal(0, 1) | 0.04 | 0.10 | 0.00 | 0.25 |
| (1+Subjectivity\|subject) | normal(0, 5) | 0.03 | 0.14 | 0.02 | 0.29 |
|  | normal(0, 2) | 0.07 | 0.14 | 0.02 | 0.29 |
|  | normal(0, 1) | 0.13 | 0.13 | 0.02 | 0.28 |
| gender: subjectivity | Normal(0, 5) | 0.005 | -0.00 | -0.17 | 0.17 |
|  | Normal(0, 10) | 0.002 | -0.00 | -0.17 | 0.17 |
|  | Normal(0, 15) | 0.002 | -0.00 | -0.18 | 0.17 |
| **subjectivity** | **Normal(0, 0.1)** | **2.00** | **-0.08** | **-0.16** | **0.01** |
|  | **Normal(0, 0.3)** | **1.01** | **-0.09** | **-0.19** | **0.01** |
|  | **Normal(0, 0.5)** | **0.62** | **-0.09** | **-0.19** | **0.00** |
| gender | Normal(0, 5) | 0.04 | -0.12 | -0.39 | 0.15 |
|  | Normal(0, 10) | 0.02 | -0.12 | -0.39 | 0.16 |
|  | Normal(0, 15) | 0.01 | -0.12 | -0.39 | 0.15 |

Table 10

*F0*

a. C1f0Maxst

| Model terms | Prior | *BF_10_* | Posterior | | |
| --- | --- | --- | --- | --- | --- |
|  |  |  | Estimate | Lower | Upper |
| (1+Subjectivity\|item) | normal(0, 10) | 0.05 | 0.35 | 0.02 | 0.84 |
|  | normal(0, 5) | 0.10 | 0.35 | 0.02 | 0.84 |
|  | normal(0, 2) | 0.26 | 0.34 | 0.01 | 0.83 |
| (1+Subjectivity\|subject) | normal(0, 10) | 0.03 | 0.23 | 0.01 | 0.63 |
|  | normal(0, 5) | 0.05 | 0.23 | 0.01 | 0.63 |
|  | normal(0, 2) | 0.14 | 0.23 | 0.01 | 0.63 |
| gender: subjectivity | Normal(0, 1) | 0.26 | -0.05 | -0.56 | 0.46 |
|  | Normal(0, 1.5) | 0.17 | -0.05 | -0.58 | 0.48 |
|  | Normal(0, 2) | 0.14 | -0.05 | -0.57 | 0.47 |
| subjectivity | Normal(0, 1) | 0.46 | 0.22 | -0.08 | 0.52 |
|  | Normal(0, 1.5) | 0.31 | 0.23 | -0.09 | 0.54 |
|  | Normal(0, 2) | 0.23 | 0.23 | -0.09 | 0.54 |
| gender | Normal(0, 15) | >>100 | -10.24 | -12.89 | -7.61 |
|  | Normal(0, 20) | >>100 | -10.25 | -12.88 | -7.63 |
|  | Normal(0, 25) | >>100 | -10.28 | -12.91 | -7.61 |

b. C1f0Minst

| Model terms | Prior | *BF_10_* | Posterior | | |
| --- | --- | --- | --- | --- | --- |
|  |  |  | Estimate | Lower | Upper |
| (1+Subjectivity\|item) | normal(0, 10) | 0.08 | 0.59 | 0.03 | 1.24 |
|  | normal(0, 5) | 0.15 | 0.59 | 0.03 | 1.23 |
|  | normal(0, 2) | 0.38 | 0.57 | 0.03 | 1.23 |
| (1+Subjectivity\|subject) | normal(0, 10) | 0.03 | 0.21 | 0.01 | 0.55 |
|  | normal(0, 5) | 0.06 | 0.21 | 0.01 | 0.55 |
|  | normal(0, 2) | 0.15 | 0.20 | 0.01 | 0.54 |
| gender: subjectivity | Normal(0, 1) | 0.27 | -0.04 | -0.56 | 0.48 |
|  | Normal(0, 1.5) | 0.18 | -0.04 | -0.57 | 0.49 |
|  | Normal(0, 2) | 0.13 | -0.04 | -0.57 | 0.50 |
| subjectivity | Normal(0, 1) | 0.22 | 0.06 | -0.34 | 0.46 |
|  | Normal(0, 1.5) | 0.14 | 0.06 | -0.35 | 0.47 |
|  | Normal(0, 2) | 0.11 | 0.06 | -0.35 | 0.48 |
| gender | Normal(0, 15) | >>100 | -12.30 | -14.09 | -10.48 |
|  | Normal(0, 20) | >>100 | -12.31 | -14.13 | -10.50 |
|  | Normal(0, 25) | >>100 | -12.31 | -14.09 | -10.53 |

c. C2f0Maxst

| Model terms | Prior | *BF_10_* | Posterior | | |
| --- | --- | --- | --- | --- | --- |
|  |  |  | Estimate | Lower | Upper |
| (1+Subjectivity\|item) | normal(0, 5) | 0.19 | 0.56 | 0.03 | 1.25 |
|  | normal(0, 2) | 0.44 | 0.55 | 0.03 | 1.23 |
|  | normal(0, 1) | 0.75 | 0.51 | 0.02 | 1.16 |
| (1+Subjectivity\|subject) | normal(0, 10) | 0.43 | 0.58 | 0.12 | 1.14 |
|  | normal(0, 5) | 0.90 | 0.59 | 0.12 | 1.16 |
|  | normal(0, 2) | 2.09 | 0.57 | 0.11 | 1.12 |
| gender: subjectivity | Normal(0, 1) | 1.40 | 0.57 | -0.09 | 1.24 |
|  | Normal(0, 1.5) | 1.09 | 0.61 | -0.07 | 1.28 |
|  | Normal(0, 2) | 0.88 | 0.62 | -0.07 | 1.31 |
| subjectivity | Normal(0, 1) | 19.46 | 0.69 | 0.24 | 1.13 |
|  | Normal(0, 1.5) | 15.17 | 0.71 | 0.26 | 1.15 |
|  | Normal(0, 2) | 12.21 | 0.72 | 0.28 | 1.16 |
| gender | Normal(0, 15) | >>100 | -10.50 | -12.19 | -8.76 |
|  | Normal(0, 20) | >>100 | -10.52 | -12.22 | -8.79 |
|  | Normal(0, 25) | >>100 | -10.53 | -12.24 | -8.82 |

d. C2f0Minst

| Model terms | Prior | *BF_10_* | Posterior | | |
| --- | --- | --- | --- | --- | --- |
|  |  |  | Estimate | Lower | Upper |
| (1+Subjectivity\|item) | normal(0, 5) | 0.16 | 0.46 | 0.02 | 1.04 |
|  | normal(0, 2) | 0.36 | 0.46 | 0.02 | 1.04 |
|  | normal(0, 1) | 0.69 | 0.43 | 0.02 | 0.99 |
| (1+Subjectivity\|subject) | normal(0, 5) | 0.04 | 0.15 | 0.01 | 0.46 |
|  | normal(0, 2) | 0.10 | 0.15 | 0.01 | 0.45 |
|  | normal(0, 1) | 0.19 | 0.15 | 0.01 | 0.44 |
| gender: subjectivity | Normal(0, 1) | 0.31 | 0.07 | -0.50 | 0.64 |
|  | Normal(0, 1.5) | 0.21 | 0.07 | -0.51 | 0.66 |
|  | Normal(0, 2) | 0.16 | 0.08 | -0.52 | 0.67 |
| subjectivity | Normal(0, 1) | 0.59 | -0.28 | -0.65 | 0.09 |
|  | Normal(0, 1.5) | 0.42 | -0.29 | -0.66 | 0.09 |
|  | Normal(0, 2) | 0.32 | -0.29 | -0.66 | 0.09 |
| gender | Normal(0, 15) | >>100 | -11.97 | -14.38 | -9.54 |
|  | Normal(0, 20) | >>100 | -12.02 | -14.48 | -9.55 |
|  | Normal(0, 25) | >>100 | -12.02 | -14.49 | -9.57 |

Table 11

*Articulation rate*

a. C1AR

| Model terms | Prior | *BF_10_* | Posterior | | |
| --- | --- | --- | --- | --- | --- |
|  |  |  | Estimate | Lower | Upper |
| (1+Subjectivity\|item) | normal(0, 5) | 0.004 | 1.07 | 0.04 | 2.06 |
|  | normal(0, 2) | 0.0005 | 0.92 | 0.03 | 1.98 |
|  | normal(0, 1) | 2.63 | 0.71 | 0.02 | 1.81 |
| (1+Subjectivity\|subject) | normal(0, 5) | 0.01 | 0.06 | 0.00 | 0.18 |
|  | normal(0, 2) | 0.04 | 0.06 | 0.00 | 0.18 |
|  | normal(0, 1) | 0.08 | 0.06 | 0.00 | 0.18 |
| gender: subjectivity | Normal(0, 0.1) | 0.78 | 0.03 | -0.11 | 0.17 |
|  | Normal(0, 0.3) | 0.40 | 0.06 | -0.14 | 0.25 |
|  | Normal(0, 0.5) | 0.25 | 0.06 | -0.14 | 0.27 |
| subjectivity | Normal(0, 1) | 0.28 | -0.01 | -0.57 | 0.58 |
|  | Normal(0, 1.5) | 0.19 | -0.01 | -0.62 | 0.60 |
|  | Normal(0, 2) | 0.15 | -0.01 | -0.60 | 0.60 |
| gender | Normal(0, 1) | 0.47 | 0.25 | -0.42 | 0.90 |
|  | Normal(0, 1.5) | 0.33 | 0.27 | -0.43 | 0.96 |
|  | Normal(0, 2) | 0.24 | 0.28 | -0.41 | 0.99 |

b. C2AR

| Model terms | Prior | *BF_10_* | Posterior | | |
| --- | --- | --- | --- | --- | --- |
|  |  |  | Estimate | Lower | Upper |
| (1+Subjectivity\|item) | normal(0, 5) | 0.13 | 0.88 | 0.03 | 1.77 |
|  | normal(0, 2) | 0.04 | 0.86 | 0.03 | 1.75 |
|  | normal(0, 1) | 0.29 | 0.68 | 0.02 | 1.61 |
| (1+Subjectivity\|subject) | normal(0, 5) | 0.03 | 0.12 | 0.01 | 0.30 |
|  | normal(0, 2) | 0.08 | 0.12 | 0.01 | 0.30 |
|  | normal(0, 1) | 0.16 | 0.12 | 0.01 | 0.30 |
| gender: subjectivity | Normal(0, 0.1) | 1.21 | -0.07 | -0.21 | 0.07 |
|  | Normal(0, 0.3) | 0.85 | -0.13 | -0.33 | 0.06 |
|  | Normal(0, 0.5) | 0.55 | -0.14 | -0.35 | 0.06 |
| subjectivity | Normal(0, 1) | 0.36 | -0.20 | -0.69 | 0.30 |
|  | Normal(0, 1.5) | 0.24 | -0.20 | -0.72 | 0.32 |
|  | Normal(0, 2) | 0.18 | -0.20 | -0.72 | 0.31 |
| gender | Normal(0, 1) | 0.43 | 0.19 | -0.60 | 0.98 |
|  | Normal(0, 1.5) | 0.31 | 0.21 | -0.62 | 1.05 |
|  | Normal(0, 2) | 0.24 | 0.22 | -0.65 | 1.06 |

Table 12

*F0 contours of the first clause*

a. FPC1

| Model terms | Prior | *BF_10_* | Posterior | | |
| --- | --- | --- | --- | --- | --- |
|  |  |  | Estimate | Lower | Upper |
| (1+Subjectivity\|item) | normal(0, 5) | 1.26 | 4.60 | 0.20 | 11.80 |
|  | normal(0, 2) | 1.05 | 1.68 | 0.06 | 4.73 |
|  | normal(0, 1) | 1.00 | 0.81 | 0.03 | 2.30 |
| (1+Subjectivity\|subject) | normal(0, 5) | 0.69 | 2.77 | 0.10 | 7.94 |
|  | normal(0, 2) | 0.91 | 1.47 | 0.06 | 4.10 |
|  | normal(0, 1) | 0.98 | 0.78 | 0.03 | 2.20 |
| gender: subjectivity | Normal(0, 5) | 0.76 | -0.73 | -8.21 | 6.66 |
|  | Normal(0, 10) | 0.54 | -1.33 | -11.26 | 8.74 |
|  | Normal(0, 15) | 0.38 | -1.56 | -12.37 | 9.25 |
| subjectivity | Normal(0, 1) | 0.96 | -0.10 | -2.00 | 1.80 |
|  | Normal(0, 1.5) | 0.94 | -0.22 | -2.93 | 2.51 |
|  | Normal(0, 2) | 0.88 | -0.34 | -3.81 | 3.14 |
| gender | Normal(0, 5) | 1.09 | -2.27 | -11.47 | 6.91 |
|  | Normal(0, 10) | 1.12 | -6.35 | -21.28 | 9.17 |
|  | Normal(0, 15) | 1.13 | -9.60 | -28.29 | 9.64 |

b. FPC2

| Model terms | Prior | *BF_10_* | Posterior | | |
| --- | --- | --- | --- | --- | --- |
|  |  |  | Estimate | Lower | Upper |
| (1+Subjectivity\|item) | normal(0, 5) | 0.89 | 3.61 | 0.14 | 10.57 |
|  | normal(0, 2) | 0.96 | 1.55 | 0.07 | 4.38 |
|  | normal(0, 1) | 0.97 | 0.79 | 0.03 | 2.22 |
| (1+Subjectivity\|subject) | normal(0, 5) | 0.92 | 3.10 | 0.14 | 7.92 |
|  | normal(0, 2) | 1.05 | 1.63 | 0.07 | 4.39 |
|  | normal(0, 1) | 1.02 | 0.82 | 0.03 | 2.27 |
| gender: subjectivity | Normal(0, 20) | 0.30 | 3.34 | -5.98 | 12.46 |
|  | Normal(0, 30) | 0.20 | 3.45 | -6.00 | 12.81 |
|  | Normal(0, 50) | 0.12 | 3.51 | -5.92 | 12.94 |
| subjectivity | Normal(0, 5) | 0.72 | -0.19 | -7.36 | 6.94 |
|  | Normal(0, 10) | 0.47 | -0.26 | -9.55 | 8.94 |
|  | Normal(0, 15) | 0.33 | -0.41 | -10.12 | 9.62 |
| gender | Normal(0, 20) | 3.21 | -13.98 | -26.78 | -0.78 |
|  | Normal(0, 30) | 2.51 | -14.77 | -28.23 | -1.24 |
|  | Normal(0, 50) | 1.66 | -15.26 | -28.76 | -1.84 |

c. FPC3

| Model terms | Prior | *BF_10_* | Posterior | | |
| --- | --- | --- | --- | --- | --- |
|  |  |  | Estimate | Lower | Upper |
| (1+Subjectivity\|item) | normal(0, 5) | 0.97 | 4.00 | 0.14 | 11.07 |
|  | normal(0, 2) | 0.98 | 1.57 | 0.06 | 4.47 |
|  | normal(0, 1) | 1.00 | 0.79 | 0.03 | 2.21 |
| (1+Subjectivity\|subject) | normal(0, 5) | 0.96 | 0.77 | 0.03 | 2.16 |
|  | normal(0, 2) | 0.96 | 0.77 | 0.03 | 2.17 |
|  | normal(0, 1) | 0.97 | 0.77 | 0.03 | 2.16 |
| gender: subjectivity | Normal(0, 20) | 1.41 | -8.57 | -17.33 | 0.17 |
|  | Normal(0, 30) | 0.99 | -8.85 | -17.76 | 0.06 |
|  | Normal(0, 50) | 0.64 | -9.04 | -18.00 | -0.14 |
| subjectivity | Normal(0, 5) | 0.70 | 2.02 | -3.12 | 7.16 |
|  | Normal(0, 10) | 0.41 | 2.53 | -3.23 | 8.33 |
|  | Normal(0, 15) | 0.29 | 2.63 | -3.32 | 8.53 |
| gender | Normal(0, 10) | 0.50 | 0.11 | -9.95 | 10.46 |
|  | Normal(0, 15) | 0.37 | 0.17 | -11.04 | 11.34 |
|  | Normal(0, 20) | 0.28 | 0.18 | -11.37 | 11.65 |
