# Supplementary material for: The Role of Prosody in Expressing Subjective and Objective Causality in English
Source: Lang Speech. 2025 Nov 2;69(3):753–77. doi: 10.1177/00238309251369482 (PMC13494113; doi:10.1177/00238309251369482)
Supplement: Supplementary file 3 [file sj-docx-3-las-10.1177_00238309251369482.docx]

# Items

**A. Experimental items used in Experiment 1 (a: objective causality; b: subjective causality; contexts in italic; target sentences in bold):**

1.

a. *You and Jim are friends. You two just talked on the phone. Now you know some information about him.*

**Jim bled a lot because he got his nose pierced.**

b. *You and Jim go to the same school, but you don’t really know him. You have the following impression of him.*

**Jim wants attention because he got his nose pierced.**

2.

a. *You are Alan’s mentor. He came to you and told you something about himself. Now you know some information about him.*

**Alan went to AA meetings because he had a drinking problem.**

b. *You and Alan used to be friends, but you haven’t talked for a long time. You don’t know for sure but you think something went wrong with him.*

**Alan can’t get his life together because he had a drinking problem**.

3.

a. *Tom is your classmate. He just messaged you about the accident he just had. Now you know some information about him.*

**Tom broke his arm because he tripped over his shoelaces.**

b. *Tom is your classmate. You don’t know him very well, but you are not very fond of him. You hold the following opinion about him.*

**Tom is an idiot because he tripped over his shoelaces.**

4.

a. *Susan is your colleague. You just met her in the hallway. She told you what just happened to her. Now you know that she had a pretty bad day.*

**Susan lost her money because she left her wallet on the bus.**

b. *Susan is your colleague. You don’t know her very well, but you have some personal opinions about her.*

**Susan is scatterbrained because she left her wallet on the bus.**

5.

a. *You came across an old friend, Tom. You two talked for a while. Now you know some information about him.*

**Tom moved out of his villa because he was bankrupt.**

b. *Tom is a businessman. You don’t know him personally, but you read from the news that he was bankrupt. You have some comments on this.*

**Tom knows nothing about business because he was bankrupt.**

6.

a. *You were in a meeting with your boss. He told you he made some decision about your colleague, Jack. Now you know the following some information.*

**Jack was fired because he was late for work every day.**

b. *You were in a meeting with your boss. You were asked to comment on your colleague, Jack. You know he is always late for work, but you don’t really know why. Here is your own guess.*

**Jack is not a morning person because he is late for work every day.**

7.

a. *Marie is your colleague. You and Marie went boating together. An accident happened to Marie. Here is what you saw with your own eyes.*

**Marie fell into the water because the boat spun around on the current.**

b. *You and your colleagues went boating together. There is one colleague, Marie, that you don’t know very well, but you have something to say about her rowing skills.*

**Marie is an inexperienced rower because the boat spun around on the current.**

8.

a. *You and Heidi are good friends. She participated in an art festival. You were there with her. Here is what you saw with your own eyes.*

**Heidi was thrilled because she won the first prize at the art festival.**

b. *Heidi is your friend. She won the first prize at the art festival. You have some comments on her success.*

**Heidi is talented because she won first prize at the art festival.**

9.

a. *Hans is your friend. You just talked with him. He told you what happened to him lately.*

**Hans bought a new house because he had won the lottery.**

b. *Hans is your friend. You think he is the luckiest person you ever know.*

**Hans is lucky because he had won the lottery.**

10.

a. *Ian is your friend. You just talked to him. He told you what he did after school yesterday.*

**Ian went to basketball practice because he was chosen for the school team.**

b. *Ian is your friend. He just told you that he was chosen for the school team of basketball. You have made some comment on his success.*

**Ian is a talented basketball player because he was chosen for the school team.**

11.

a. *Joshua is your friend. You two just talked on the phone. Now you know some information from him.*

**Joshua's fingers hurt because he had played the guitar for hours.**

b. *Joshua is your neighbor. He has been playing guitar for the whole afternoon. You don’t know Joshua in person but you guess the following.*

**Joshua has a great passion for music because he has played the guitar for hours.**

12.

a. *Julia is your sister. You know that her old shelf is too small to fit all her books. She bought a new one.*

**Julia bought a new shelf because she has a lot of books.**

b. *Julia is your classmate. You know that she has a lot of books. You guess the following.*

**Julia loves reading because she has a lot of books.**

13.

a. *You got the card on the left from your friend. On it you can see the following things.*

**The girl is laughing because the kite is flying.**

b. *You got a card from a friend. It has a picture of a flying kite on it. You are asked to make predictions on what makes the kite fly. Here is what you came up with.*

**The wind is blowing because the kite is flying.**

14.

a. *Jonny is your friend. You two are very close. You know everything about him.*

**Jonny works as a mover because he can lift a sofa easily.**

b. *From the picture you can see a man. You have made some predictions based on what you saw. Here is what you came up with.*

**The man is strong because he can lift a sofa easily.**

15.

a. *From the picture on the left you learned the following information.*

**The boy is covered with mud because he is playing in the mud.**

b. *From the picture on the left you can see a boy playing in the mud. You come up with the following comments based on what you saw.*

**The boy likes mud because he is playing in the mud.**

**B. Experimental items used in Experiment 2 (a: objective causality; b: subjective causality; contexts in italic; target sentences in bold):**

1.

a. *You and Jim are friends. You two just talked on the phone. Now you know some information about him.*

**Jim got his nose pierced so he bled a lot.**

b. *You and Jim go to the same school, but you don’t really know him. You have the following impression of him.*

**Jim got his nose pierced so he wants attention.**

2.

a. *You are Alan’s mentor. He came to you and told you something about himself. Now you know some information about him.*

**Alan has a drinking problem so he goes to AA meetings.**

b. *You and Alan used to be friends, but you haven’t talked for a long time. You don’t know for sure but you think something went wrong with him.*

**Alan has a drinking problem so he has weak self-control.**

3.

a. *Tom is your classmate. He just messaged you about the accident he just had. Now you know some information about him.*

**Tom tripped over his shoelaces so he broke his arm.**

b. *Tom is your classmate. You don’t know him very well, but you are not very fond of him. You hold the following opinion about him.*

**Tom tripped over his shoelaces so he is an idiot.**

4.

a. *Susan is your colleague. You just met her in the hallway. She told you what just happened to her. Now you know that she had a pretty bad day.*

**Susan left her wallet on the bus so she lost her money.**

b. *Susan is your colleague. You don’t know her very well, but you have some personal opinions about her.*

**Susan left her wallet on the bus so she is scatterbrained.**

5.

a. *You came across an old friend, Tom. You two talked for a while. Now you know some information about him.*

**Tom went bankrupt so he had to move out of his villa.**

b. *Tom is a businessman. You don’t know him personally, but you read from the news that he was bankrupt. You have some comments on this.*

**Tom went bankrupt so he knew nothing about business.**

6.

a. *You were in a meeting with your boss. He told you he made some decision about your colleague, Jack. Now you know the following information.*

**Jack was always late for work so he was fired.**

b. *You were in a meeting with your boss. You were asked to comment on your colleague, Jack. You know he is always late for work, but you don’t really know why. Here is your own guess.*

**Jack is always late for work so he is not a morning person.**

7.

a. *Marie is your colleague. You and Marie went boating together. An accident happened to Marie. Here is what you saw with your own eyes.*

**The boat spun around on the current so Marie fell into the water.**

b. *You and your colleagues went boating together. There is one colleague, Marie, that you don’t know very well, but you have something to say about her rowing skills.*

**The boat spun around on the current so Marie doesn’t know much about rowing.**

8.

a. *You and Heidi are good friends. She participated in an art festival. You were there with her. Here is what you saw with your own eyes.*

**Heidi won the first prize at the art festival so she was happy.**

b. *Heidi is your good friend. She won the first prize at the art festival. You have some comments on her success.*

**Heidi won the first prize at the art festival so she is talented.**

9.

a. *Hans is your friend. You just talked with him. He told you what happened to him lately.*

**Hans won the lottery so he bought a new house.**

b. Hans is your friend. You think he is the luckiest person you ever know.

**Hans won the lottery so he is lucky.**

10.

a. *Ian is your friend. You just talked to him. He told you what he did after school yesterday.*

**Ian made the school team so he went to basketball practice.**

b. *Ian is your friend. He just told you that he was chosen for the school team of basketball. You have made some comment on his success.*

**Ian made the school team so he is a talented basketball player.**

11.

a. *Joshua is your friend. You two just talked on the phone. Now you know some information from him.*

**Joshua played the guitar for hours so his fingers hurt.**

b. *Joshua is your neighbor. He has been playing guitar for the whole afternoon. You don’t know Joshua in person but you guess the following.*

**Joshua played the guitar for hours so he has a great passion for music.**

12.

a. *Julia is your sister. You just talked to her. Now you know the following information from her.*

**Julia has so many books so she gave some away.**

b. *Julia is your classmate. You know that she has a lot of books. You guess the following.*

**Julia has so many books so she loves reading.**

13.

a. *You got the card on the left from your friend. On it you can see the following things.*

**The kite is flying so the girl is laughing.**

b. *You got a card from a friend. There is a flying kite on it. You are asked to make predictions on what makes the kite fly. Here is what you came up with.*

**The kite is flying so the wind is blowing.**

14.

a. *Jonny is your friend. You two are very close. You know almost everything about him.*

**Jonny can lift a sofa easily so he helped his friend move house.**

b. *From the picture you can see a man. You have some comments about what you saw. Here is what you came up with.*

**The man can lift a sofa easily so he is strong.**

15.

a. *From the picture on the left you learn the following information.*

**The boy is playing in the mud so he gets covered in it.**

b. *From the picture on the left you can see a boy playing in the mud. You come up with the following comments based on what you saw.*

**The boy is playing in the mud so he likes mud.**

**C. Filler items, Experiments 1 and 2 (note: the connectives were *however* in Experiment 1)**

1f.

*You met your colleague, Ben, during coffee break. You two talked for a while. Now you know what he went through last evening.*

**Ben locked himself out of his own house, but he managed to get in.**

2f.

*You and John are friends. He told you what he has gone through recently. Here is what he told you.*

**John worked very hard, but he failed his exams.**

3f.

*You just talked on the phone with your brother, Leo. Now you know something about his business trip to Paris.*

**Leo went to Paris, but he didn’t visit the Eiffel Tower.**

4f.

*You met your colleague, Bill, in the hallway. You two talked for a while. He told you how he spent his weekend.*

**Bill is a big fan of basketball, but he missed the all-star game yesterday.**

5f.

*Jessica is your friend. You two chatted for a while. Jessica told you about how her life’s been going recently. Now you know the following information.*

**The new “Star Wars” is nice, but Jessica hasn’t watched it.**

6f.

*Alan is your roommate. You know him quite well. Here is something you know about him.*

**Alan was born in China, but he doesn’t speak Mandarin.**

7f.

*You are Tim’s boss. You two have been working together for years. You are quite familiar with him. Here is something that you know about him.*

**Tim is healthy as a horse, but he called in sick today.**

8f.

*You are taking a walk in the park. You saw your neighbor’s kid, Mark, play with other kids on the ground. And then the following things happened.*

**Mark tripped over a rock and scratched his knee, but he did not cry.**

9f.

*You and your friend, Lisa, were chatting on the phone. Lisa mentioned her neighbor, Jane. Now you know something about Jane.*

***Jane and Lisa are neighbors, but they rarely see each other.***

10f.

*You came cross a friend, Lily. You two talked for a while. Now you know something about her trip to London.*

**Lily’s flight departed later than scheduled, but the plane landed on time.**

11f.

*You and Sam are friends. You two chatted on the phone. He told you something about his assignment.*

**The math paper was difficult, but Sam finished it on his own.**

12f.

*You ran into an old friend, Rose. She told you about the dress she saw in the mall. Now you know the following information.*

**Rose liked the dress very much, but she decided not to buy it.**

13f.

*You and Dylan are friends. You two are catching up on the phone. You talked about his project. Now you know the following information.*

**Dylan had planned his project carefully, but the project didn’t go very well.**

14f.

*Your colleague, David, just got back from his lunch. He told you how lucky he was in the café. Now you know the following information.*

**The café was crowded, but David found a table.**

15f.

*Sam works for you as your assistant. He updated you about the report he has been working on. Now you know about his progress.*

**The report was already proofread, but Sam found additional mistakes in it.**

16f.

*Andy is your brother. You two just talked on WhatsApp. Now you know about his trip in Budapest.*

**The weather was bad, but Andy had a good time.**

17f.

*You and Lisa are talking about your common friend, Alex. Lisa told you the following information about Alex.*

**Alex didn’t feel very well, but she didn’t go to see the doctor.**

18f.

*Benjamin is your officemate. You know him quite well. Here is something you know about him.*

**Benjamin has a car, but he doesn’t drive it very often.**

19f.

*You and Becky are close friends. She told you about her job offer. Now you know the following information.*

**The job is not well-paid, but Becky wants to take it.**

20f.

*Andy is your colleague. You have been working together for years. You know him quite well.*

**Andy is usually very punctual, but he was late for the meeting this morning.**
